# *VPS13B*, gene responsible for Cohen syndrome, regulates gingival epithelial barrier function via intracellular trafficking of coxsackievirus and adenovirus receptor

**DOI:** 10.1038/s41598-026-40840-9

**Published:** 2026-02-24

**Authors:** Risako Matsumura, Keita Tanigaki, Naoko Sasaki, Tsukasa Tamamori, Shunsuke Yamaga, Akito Sakanaka, Atsuo Amano, Michiya Matsusaki, Hiroki Takeuchi, Masae Kuboniwa

**Affiliations:** 1 Department of Preventive Dentistry, Graduate School of Dentistry, The Osaka University, Suita, Osaka 565-0871 Japan; 2 Department of Preventive Dentistry, The Osaka University Dental Hospital, 1-8 Yamadaoka, Suita, Osaka 565-0871 Japan; 3 Joint Research Laboratory (TOPPAN) for Advanced Cell Regulatory Chemistry, Graduate School of Engineering, The Osaka University, Suita, Osaka 565-0871 Japan; 4 Department of Applied Chemistry, Graduate School of Engineering, The Osaka University, Suita, Osaka 565-0871 Japan

**Keywords:** Cell biology, Molecular biology, Diseases

## Abstract

Cohen syndrome is an autosomal recessive genetic disease caused by mutations in the vacuolar protein sorting homolog B (*VPS13B*) gene that leads to a variety of complications including periodontitis. However, the molecular mechanism underlying periodontal inflammation caused by *VPS13B* dysfunction in human gingival epithelial cells remains unclear. A previous report noted that coxsackievirus and adenovirus receptor (CXADR) and junctional adhesion molecule 1 (JAM1) are involved in barrier functions against penetration by lipopolysaccharide (LPS) and peptidoglycan (PGN) into gingival tissues. The present study was conducted to examine the effects and significance of *VPS13B* on gingival barrier function. It was confirmed that loss of VPS13B resulted in decreased cell surface localization of CXADR, but not of JAM1. Additionally, abundant lysosomal localization of CXADR was detected in *VPS13B*-knockout cells followed treatment with bafilomycin A1, an inhibitor of lysosomal degradation. Other findings indicated that cell-surface localization of the CXADR-chimeric protein, in which C-terminus was exchanged with JAM1, was not disturbed by *VPS13B* knockout. Finally, *VPS13B* knockout led to greater permeability of gingival epithelial cell layers and tissues to LPS and PGN, which was restored by increased expression of CXADR-JAM1^c−term^. Together, these results show that VPS13B is involved in intracellular trafficking of CXADR as well as the barrier function of human gingival epithelial tissues, and thus indicate the molecular basis for periodontal complications in patients affected by Cohen syndrome.

**Supplementary Information:**

The online version contains supplementary material available at 10.1038/s41598-026-40840-9.

## Introduction

Cohen syndrome is an autosomal recessive genetic disease^1^, with affected individuals characterized by developmental delay, distinct craniofacial abnormalities, potential ocular dysfunction, neutropenia, and severe periodontitis^2–4^. Findings have been presented showing that pathogenic mutations in vacuolar protein sorting 13 homolog B (VPS13B) at chromosomal locus 8q22 are causative factors^5^. The VPS13B protein is known to be associated with the Golgi apparatus, and found to maintain Golgi integrity and function for vesicle-mediated transport and sorting of proteins within cells^5,6^. The Golgi apparatus is an organelle in eukaryotic cells that functions to package proteins into membrane-bound intracellular vesicles before being sent to their destinations. This process occurs at the intersection of the secretory and lysosomal pathways, and its detailed transport system is mediated by VPS13B. However, it is not well understood, especially in regard to the etiology of periodontitis.

Periodontitis is a chronic inflammatory condition that affects the periodontium, a complex structure comprised of gingiva, periodontal ligament, cementum, and alveolar bone. The etiology of periodontitis is initiated by chronic infection of gingival epithelial cells by oral commensal bacteria^7^. To understand the effects of VPS13B on host cells, the relationship of neutrophil dysfunction and progression of periodontitis in patients with genetic disorders has been investigated^8^. Nevertheless, regarding the gingival epithelial barrier, the link between VPS13B and periodontal disease has yet to be clearly elucidated. Recently, we presented findings showing that coxsackievirus and adenovirus receptor (CXADR), a tight junction-associated protein, is involved in epithelial barrier function in gingival tissues, though is degraded by the periodontal pathogen *Porphyromonas gingivalis*, which leads to penetration by lipopolysaccharide (LPS) and peptidoglycan (PGN)^9^. In this regard, CXADR is potentially vulnerable and targeted by periodontal risk factors, which led to speculation that CXADR dysfunction in gingival epithelial tissues contributes to periodontal disease onset in Cohen syndrome patients.

Findings obtained in our previous study showed that CXADR is transported via the endomembrane system^9^. Others have reported that CXADR possesses a transmembrane domain closely related to palmitoylated residues^10^ and a cytoplasmic domain with a PDZ-binding motif for interaction with proteins containing PDZ domains^11^, with each of those domains unique as compared to other tight junction-related proteins, including JAM1^12^. It is thus considered that the C-terminus from the transmembrane to cytosolic tail is potentially important for CXADR transport in the endomembrane system via the Golgi apparatus, though these mechanisms remain unclear.

Other studies have obtained findings showing that VPS13B forms a complex with RAB GTPase RAB6^13^, family with sequence similarity 177 member A1 (FAM177A1)^14^, Sect. 23 interacting protein (SEC23IP)^15^, and syntaxin 13 (STX13)^16^. Of those, RAB6 is a small GTPase protein associated with medial and trans-Golgi cisternae^17^, and the surface of the trans-Golgi network^18^, thus is involved with regulation of vesicle transport via the Golgi apparatus^13^. Additionally, FAM177A1 has been shown as a Golgi complex protein^14,19^ related to assembly of the Golgi complex and SEC23IP has been shown to be localized at the ER exit site (ERES), with tethering of VPS13B to ERES. Furthermore, STX13 is mainly localized in the endosomal region and has been reported to recruit VPS13B for regulation of vesicle transport^16^. As for VPS13B, several studies have focussed on Golgi morphology factors, though the target protein as well as trafficking mechanism regulated by VPS13B remain unknown.

We previously established living in vitro tissue models composed of human cells and extracellular matrices^9,20–23^. However, three-dimensional tissue models of epithelium enable monitoring of fluorescent materials by photometry or microscopy for evaluation of the effects of harmful factors on barrier function, as quantitative evaluation in animal models as well as humans is difficult. Cohen syndrome patients often undergo long-term antibiotic and recombinant protein therapy to prevent infection. To exclude systemic effects on gingival epithelial tissues, it is considered that genetic disorder-specific tissue models can be useful for understanding the pathogenesis of periodontitis.

The present study was conducted to assess the involvement of *VPS13B* in CXADR-mediated barrier function of gingival epithelial tissue for better understanding of the etiology of periodontal disease associated with Cohen syndrome. Genome editing was performed with the combination of clustered regularly interspaced short palindromic repeats CRISPR-associated protein 9 (CRISPR-Cas9) and short hairpin RNA (shRNA)-expression systems using a three-dimensional tissue model.

## Results

### VPS13B localization in human gingival epithelial cells

**Fig. 1:**
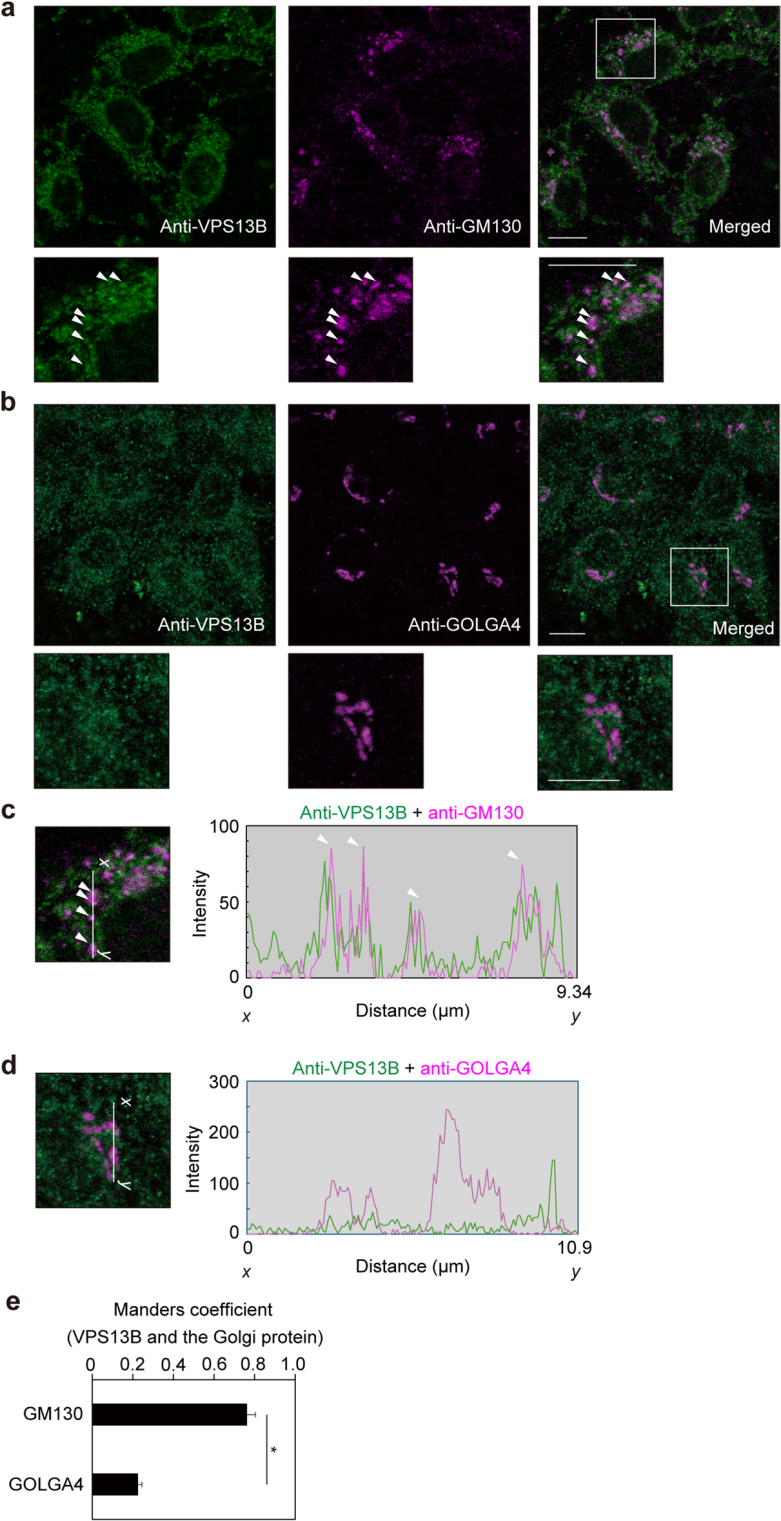
VPS13B localization in IHGE cells. (**a**, **b**) IHGE cells were fixed, stained with rabbit polyclonal anti-VPS13B (green: Alexa Fluor 488) and either of mouse monoclonal anti-GM130 (magenta in a: Alexa Fluor 555) or mouse monoclonal anti-GOLGA4 (magenta in b: Alexa Fluor 555), and analyzed by confocal microscopy. Higher magnifications of the areas indicated by white boxes in the upper panels are shown on the lower side. Scale bars, 10 μm. (**c**, **d**) The intensity (LAS X, Leica) of the fluorescence signals of VPS13B (green) and either of GM130 (magenta) or GOLGA (magenta) on the *x*-*y* lines in higher magnification in (**a**) and (**b**) are shown. (**e**) Comparison of co-efficiency between VPS13B and either of GM130 or GOLGA4 as the denominator. Values are shown as the mean ± SD of ten technical replicates. *p < 0.05, two-tailed *t* test. Data shown are representative of two biological replicates.

Previous examinations conducted to analyze VPS13B expression mainly used HeLa cell line models^13,24^, while no such studies using human gingival epithelial cells have been presented. In the present study, the distribution of VPS13B was examined using immortalized human gingival epithelial (IHGE) cells. VPS13B has been reported to colocalize with the cis-Golgi marker GM130^6^. The findings confirmed associations of VPS13B with the cis-Golgi markers GM130, while the association with GOLGA4, a trans-Golgi marker, was scant (Fig. 1a and d). Additionally, compared to GOLGA4, a higher Manders coefficient was observed between the fluorescent signals of VPS13B and GM130 (Fig. 1e). These results thus indicated that in gingival epithelial cells VPS13B is mainly localized in cis-Golgi.

### VPS13B involved in CXADR localization in gingival epithelial cells

To analyze intracellular localization of VPS13B and CXADR in gingival epithelial cells, IHGE cells were transfected with plasmid coding Myc-tagged hemagglutinin (HA)-inserted CXADR^9^ and subjected to immunocytochemistry. As shown in Supplementary Fig. 1a, the anti-Myc signal indicates the N-terminal region cleaved at signal peptide, while the anti-HA signal corresponding to the cleaved C-terminal region (mature form). Confocal microscopic analysis confirmed co-localization of endogenous VPS13B and HA-inserted CXADR, but hardly anti-Myc signals (Supplementary Fig. 1b-1d). These findings indicate an association of VPS13B and mature form of CXADR in human gingival epithelial cells.

**Fig. 2:**
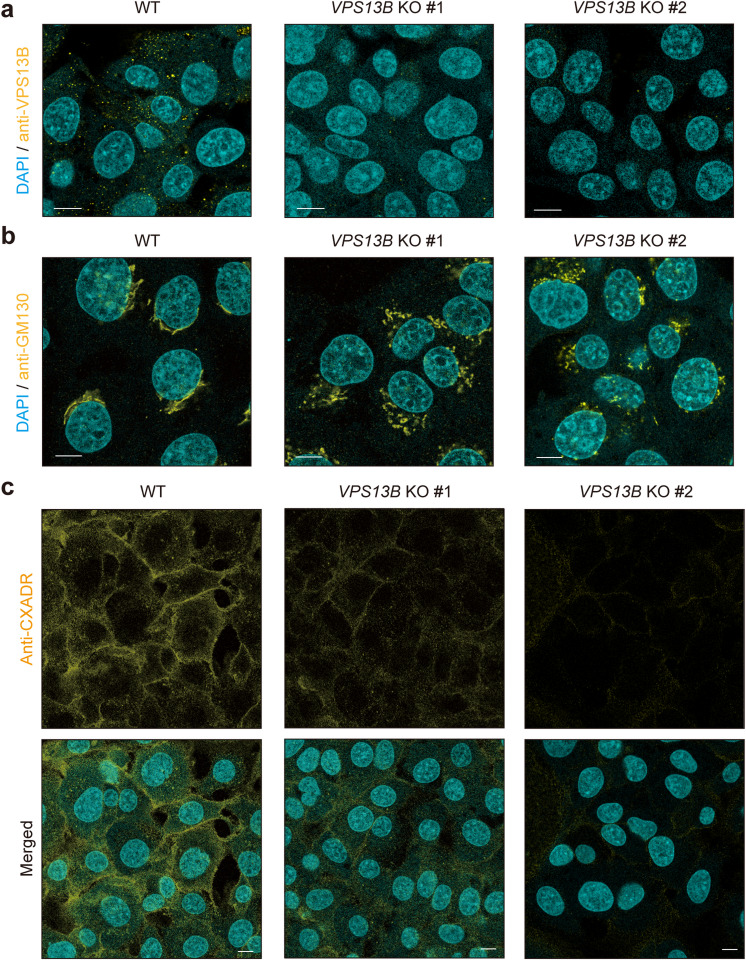
*VPS13B* KO decreases CXADR in IHGE cells. (**a**-**c**) IHGE WT, *VPS13B*-KO #1, and *VPS13B*-KO #2 cells were fixed, stained with DAPI (cyan), and either rabbit polyclonal anti-VPS13B (yellow in a: Alexa Fluor 555), mouse monoclonal anti-GM130 (yellow in b: Alexa Fluor 555), or rabbit monoclonal anti CXADR (yellow in c: Alexa Fluor 555), then analyzed using confocal microscopy. Scale bars, 10 μm. Result is representative of two biological replicates. See also Supplementary Fig. 3.

Next, the involvement of VPS13B in proteins regulating barrier function in gingival epithelial cells was analyzed. IHGE cells lacking *VPS13B* were constructed, with knockout confirmed by immunofluorescence staining (Fig. 2a) as well as reverse transcription polymerase chain reaction (RT-PCR) findings (Supplementary Fig. 2). Dispersion of GM130-positive compartments in *VPS13B* knockout (KO) cells was then confirmed (Fig. 2b). As shown in Supplementary Fig. 3a and 3b, intact Golgi morphology was observed in WT cells, but not in *VPS13B*-KO cells. On the contrary, fully fragmented morphology was observed in KO cells, but hardly in WT cells. These phenotype matches results of the previous studies^6,24^. Under this condition, decreased CXADR expression in *VPS13B-*KO cells as compared to WT cells was noted (Fig. 2c, Supplementary Fig. 3c). To exclude off-target effects of the CRISPR-Cas9 system against *VPS13B*, IHGE cells stably expressing small hairpin RNA (shRNA) against *VPS13B* (shVPS13B #1458) and firefly *luciferase* (shLuc, control) were constructed^25^ (Supplementary Fig. 4a), and the findings showed decreased CXADR on the surface of cells expressing shVPS13B (Supplementary Fig. 4b), indicating that a decrease in CXADR level by *VPS13B* KO or knockdown was not an off-target effect. Additionally, *VPS13B* KO decreased the level of protein associated with *CXADR* (Supplementary Fig. 5a and 5b), but not that of mRNA (Supplementary Fig. 5c), indicating that the post-transcriptional cascade of CXADR is disrupted by *VPS13B* KO. Findings of our previous study indicated that junctional adhesion molecule 1 (JAM1), another tight junction-related protein, is involved in the barrier function of gingival epithelial tissues^20,26^, thus the effects of *VPS13B* KO on JAM1 localization were examined and a negligible effect on JAM1 expression was confirmed (Supplementary Fig. 6). Together, these results suggest that VPS13B is selectively involved in CXADR localization in gingival epithelial cells.

### Loss of *VPS13B* causes abnormal transport of CXADR to lysosome in gingival epithelial cells

**Fig. 3:**
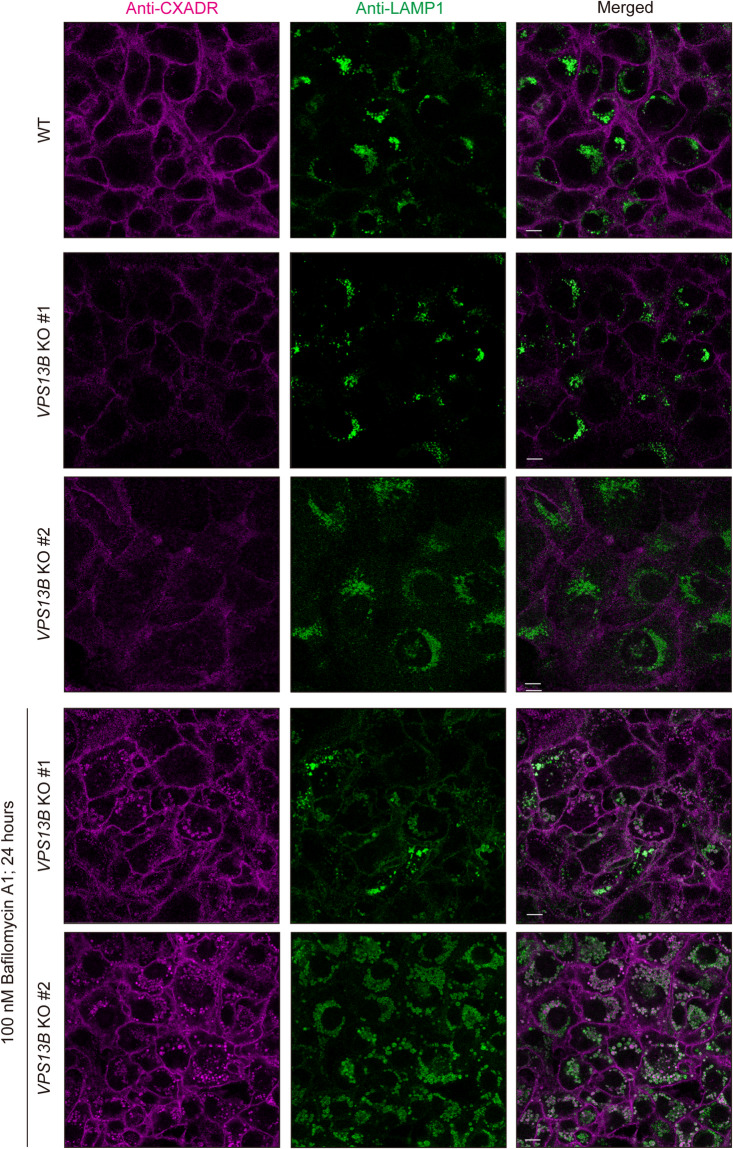
CXADR co-localization with lysosome in IHGE *VPS13B*-KO cells treated with bafilomycin A1. IHGE WT, *VPS13B*-KO #1, and *VPS13B*-KO #2 cells were treated with or without bafilomycin A1 (100 nM) for 24 h, then fixed, stained with rabbit monoclonal anti-CXADR (magenta: Alexa Fluor 647) or mouse monoclonal anti-LAMP1 (green: Alexa Fluor 555), and analyzed by confocal microscopy. Scale bars, 10 μm. Result is representative of two biological replicates. See also Supplementary Figs. 7 and 8.

The level of CXADR, a transmembrane protein transported to the cell surface via an endocytic pathway^9^, was found to be decreased by *VPS13B* KO (Supplementary Fig. 5a and 5b). It was thus hypothesized that CXADR is missorted to a host-degradation pathway that includes lysosomes. To test this hypothesis, WT and *VPS13B* KO IHGE cells were treated with bafilomycin A1, known as an inhibitor of lysosomal acidification^27^, then the association of CXADR with the lysosome marker LAMP1 was examined using confocal microscopy. As shown in Fig. 3 and Supplementary Fig. 7a and 7b, abundant co-localization of CXADR with LAMP1 in *VPS13B* KO cells was observed. Furthermore, it was noted that a decrease in CXADR on the cell surface was effectively restored in *VPS13B* KO cells by treatment with bafilomycin A1 (Supplementary Fig. 8). The autophagy-lysosomal pathway is known to degrade long-living proteins in host cells^28^. To exclude the possible effects of autophagy on VPS13B-CXADR interaction, IHGE cells were treated with EACC, an inhibitor of autophagosome-lysosome fusion^29^, then localization of CXADR and LAMP1 was analyzed, with the findings confirming negligible effects of EACC on decrease of CXADR in *VPS13B*-KO cells (Supplementary Fig. 9). These results thus indicate that CXADR degradation by *VPS13B* KO is caused by abnormal degradation of lysosomes, but not by autophagy.

### Loss of *RAB6A* and VPS13B shows common phenotypes indicating decreased CXADR in gingival epithelial cells

**Fig. 4:**
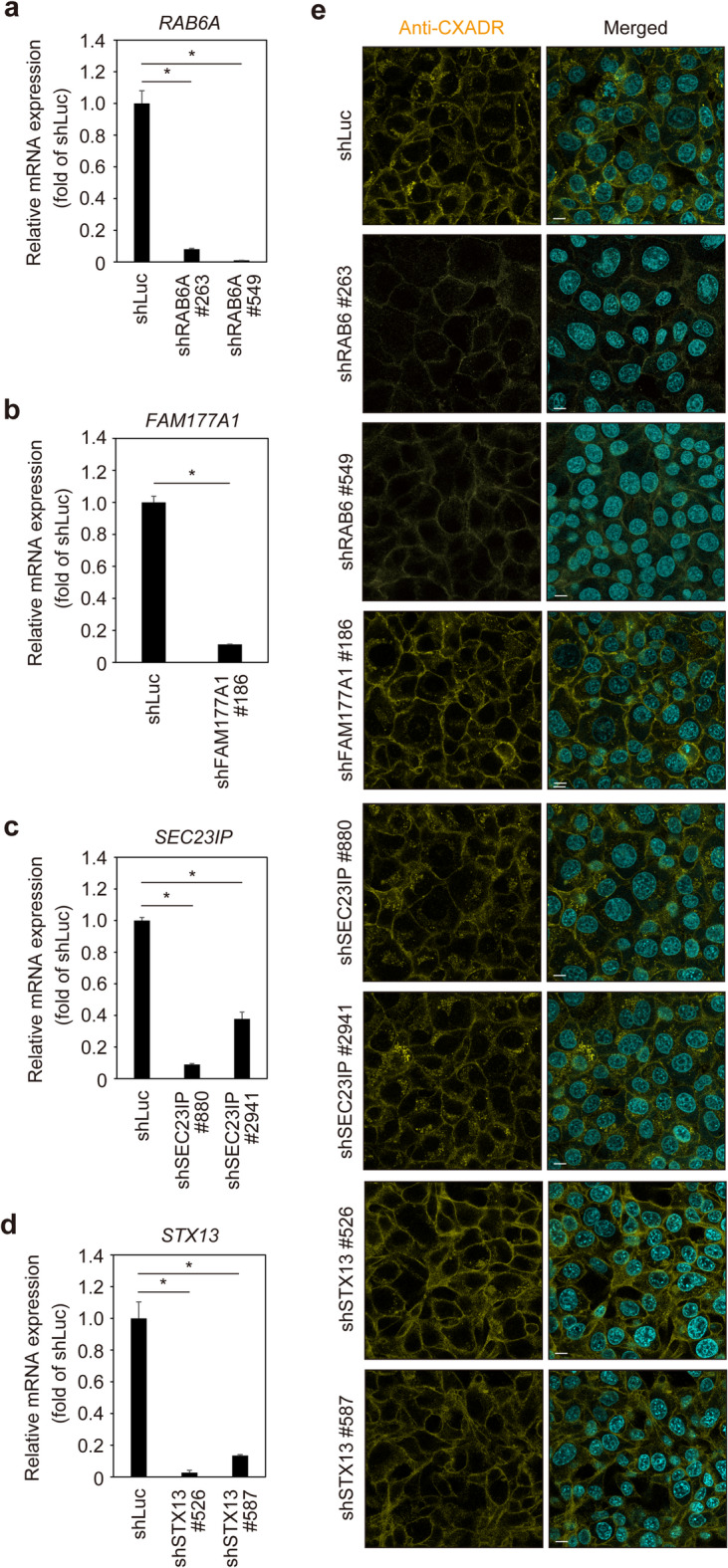
Effects of knockdown of VPS13B-related genes on CXADR localization in IHGE cells. (**a-d**) Fold change of relative mRNA expression of indicated genes in association with VPS13B (*RAB6A* [a], *FAM177A* [b], *SEC23IP* [c] and *STX13* [d]) in IHGE cells expressing shLuc or the indicated shRNA relative to *β-ACTIN* expression, indicated by mean values ± SD of three technical replicates. (**e**) IHGE cells expressing shLuc, shRAB6A (#263, #549), shFAM177A (#186), shSEC23IP (#880, #2941), or shSTX13 (#526, #587) were fixed, stained with DAPI (cyan) and rabbit monoclonal anti-CXADR (yellow: Alexa Fluor 555), and analyzed by confocal microscopy. Scale bars, 10 μm. Result is representative of three biological replicates. See also Supplementary Fig. 10.

To examine the effects of VPS13B-associated proteins on CXADR localization, IHGE cells stably expressing shRNA against *RAB6A* (Fig. 4a), *FAM177A1* (Fig. 4b), *SEC23IP* (Fig. 4c), or *STX13* (Fig. 4d) were constructed, then CXADR localization was analyzed by confocal microscopy. A decreased level of CXADR was noted in *RAB6A* knockdown cells, while negligible effects were seen in *FAM177A1*,* SEC23IP*, and *STX13* knockdown cells (Supplementary Fig. 10), with these findings confirmed using immunoblot assays following plasma-membrane fractionation (Supplementary Fig. 11a and 11b). Thus, RAB6A and VPS13B are considered to be involved in the same cascade of intracellular transport of CXADR in gingival epithelial cells.

### C-terminus from transmembrane domain of CXADR involved in VPS13B-mediated transport in gingival epithelial cells

**Fig. 5:**
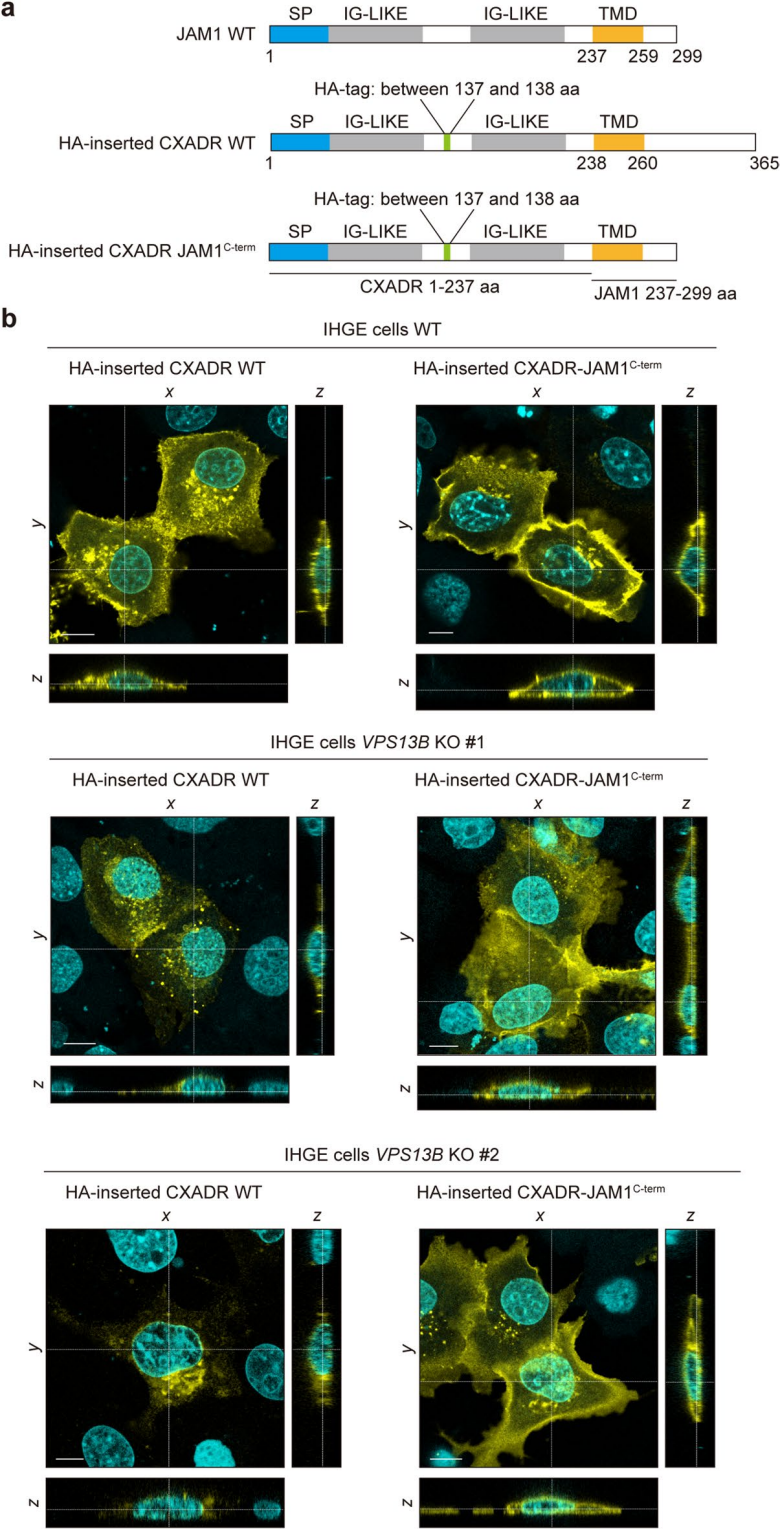
CXADR WT and JAM1^C−term^ localization in IHGE *VPS13B*-KO cells. (**a**) Schematic view of JAM1 WT, HA-inserted CXADR WT, and HA-inserted CXADR-JAM1^C-term^. SP (cyan), IG-LIKE (gray), and TMD (yellow) domains are indicated. HA-tag is shown in green. (**b**) IHGE WT, *VPS13B*-KO #1, and *VPS13B*-KO #2 cells transiently expressing HA-inserted CXADR WT or CXADR-JAM1^C-term^ were fixed, stained with DAPI (cyan) and rabbit monoclonal anti-HA (yellow: Alexa Fluor 555), and analyzed by confocal microscopy. Scale bars, 10 μm. Result is representative of two biological replicates. See also Supplementary Fig. 12.

Since RAB GTPases, including RAB6A, are localized in the cytosolic side of the organelle and JAM1 localization was negligibly altered by *VPS13B* knockout (Supplementary Fig. 6), it was hypothesized that the C-terminus of the transmembrane domain of CXADR is selectively involved in VPS13B-medaited intracellular traffic. To confirm the responsible amino acid region, an HA-inserted CXADR chimeric mutant, in which the C-terminus was replaced by that of JAM1 (CXADR-JAM1^C−term^), was constructed (Fig. 5a) and transfected into IHGE cells. Using confocal microscopy, the cell-surface signal was confirmed in WT cells expressing HA-inserted CXADR WT and CXADR-JAM1^C−term^ (Fig. 5b). In contrast, HA-inserted CXADR WT signaling was decreased in *VPS13B-*KO cells, but then restored in cells with chimeric expression of CXADR-JAM1^C−term^ (Supplementary Fig. 12a and 12b). To confirm the relevance of C-terminus from the transmembrane domain of JAM1 as well as CXADR, cells with HA-inserted JAM1 WT and JAM1-CXADR^C−term^ were also constructed (Supplementary Fig. 13a), and transfected into IHGE cells, with the signals then analyzed by confocal microscopy. In WT cells expressing HA-inserted JAM1 WT and JAM1-CXADR^C−term^, cell-surface signals were confirmed (Supplementary Fig. 13b). As for *VPS13B*-KO cells, HA-inserted JAM1 WT signals were not altered, though were decreased in those with chimeric expression of JAM1-CXADR^C−term^. These results indicate that the C-terminus from the transmembrane domain of CXADR is responsible for VPS13B-mediated transport in gingival epithelial cells.

### Epithelial barrier function regulated by VPS13B dependently of CXADR

**Fig. 6:**
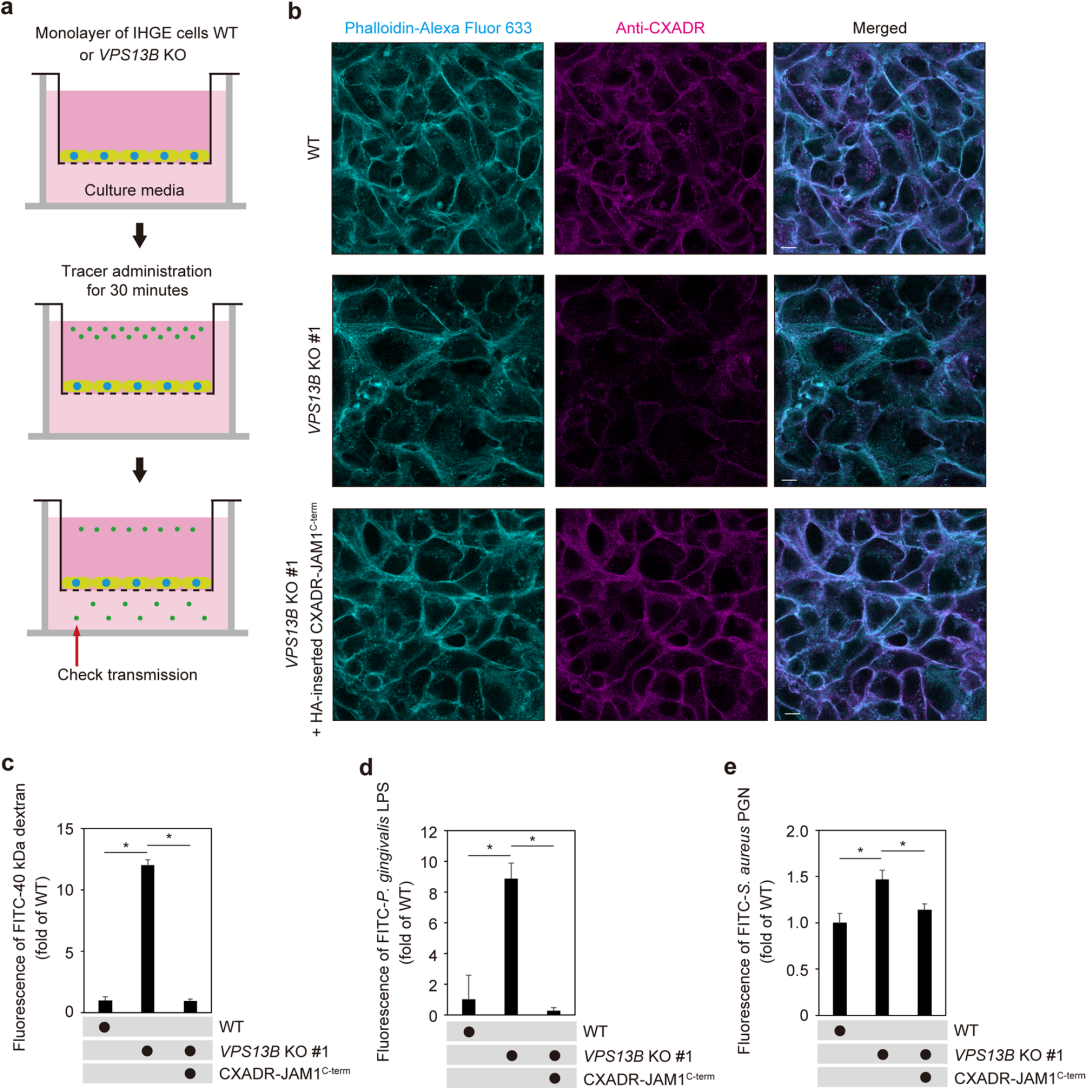
*VPS13B* KO reduces epithelial barrier function of gingival epithelial cell layer dependent on extracellular CXADR regions. (**a**) Schematic image of culture-insert system. Gingival epithelial layer WT or *VPS13B*-KO #1 cells with or without overexpression of HA-inserted CXADR-JAM1^C−term^ were cultured in the upper compartments. FITC-labeled tracers were then added to culture medium in the upper compartments. After 30 min of incubation, transmission of a tracer from the upper to lower compartment was analyzed using spectrometry. (**b**) Confocal microscopic images of IHGE cell layer. WT or *VPS13B-*KO #1 cell layers on coverslips with or without overexpression of HA-inserted CXADR-JAM1^C−term^ were fixed, stained with Alexa Fluor 633-conjugated phalloidin (cyan) and anti-CXADR (magenta), and analyzed using confocal microscopy. Scale bars, 10 μm. See also Supplementary Fig. 14. (**c-e**) Cell layer permeability to FITC–40 kDa dextran (**c**), *P. gingivalis* LPS (**d**), and *S. aureus* PGN (**e**). Results are expressed as fold change relative to the control (WT) and shown as the mean ± SD of eight technical replicates. *p < 0.05, two-tailed *t* test (closed-testing procedure). Data shown are representative of two biological replicates.

Next, a two-layered culture system was used to assess the role of VPS13B in barrier function of the gingival epithelial cell layer (Fig. 6a). *VPS13B*-KO cells additionally expressing HA-inserted CXADR-JAM1^C−term^ were generated so as to eliminate off-target effects due to the knockout system and findings obtained confirmed sufficient compensation of CXADR-chimeric protein on cell surfaces (Fig. 6b, Supplementary Figs. 14 and 15). Under this condition, permeability assay results indicated that *VPS13B* depletion increased permeation of fluorescein isothiocyanate (FITC)-labeled 40 kDa dextran (Fig. 6c), *P. gingivalis* LPS (Fig. 6d), and PGN (Fig. 6e), which was abrogated by CXADR-JAM1^C−term^ overexpression. Thus, it is considered that loss of cell-surface CXADR is involved in increased permeability of the gingival epithelial layer of *VPS13B* KO cells.

**Fig. 7:**
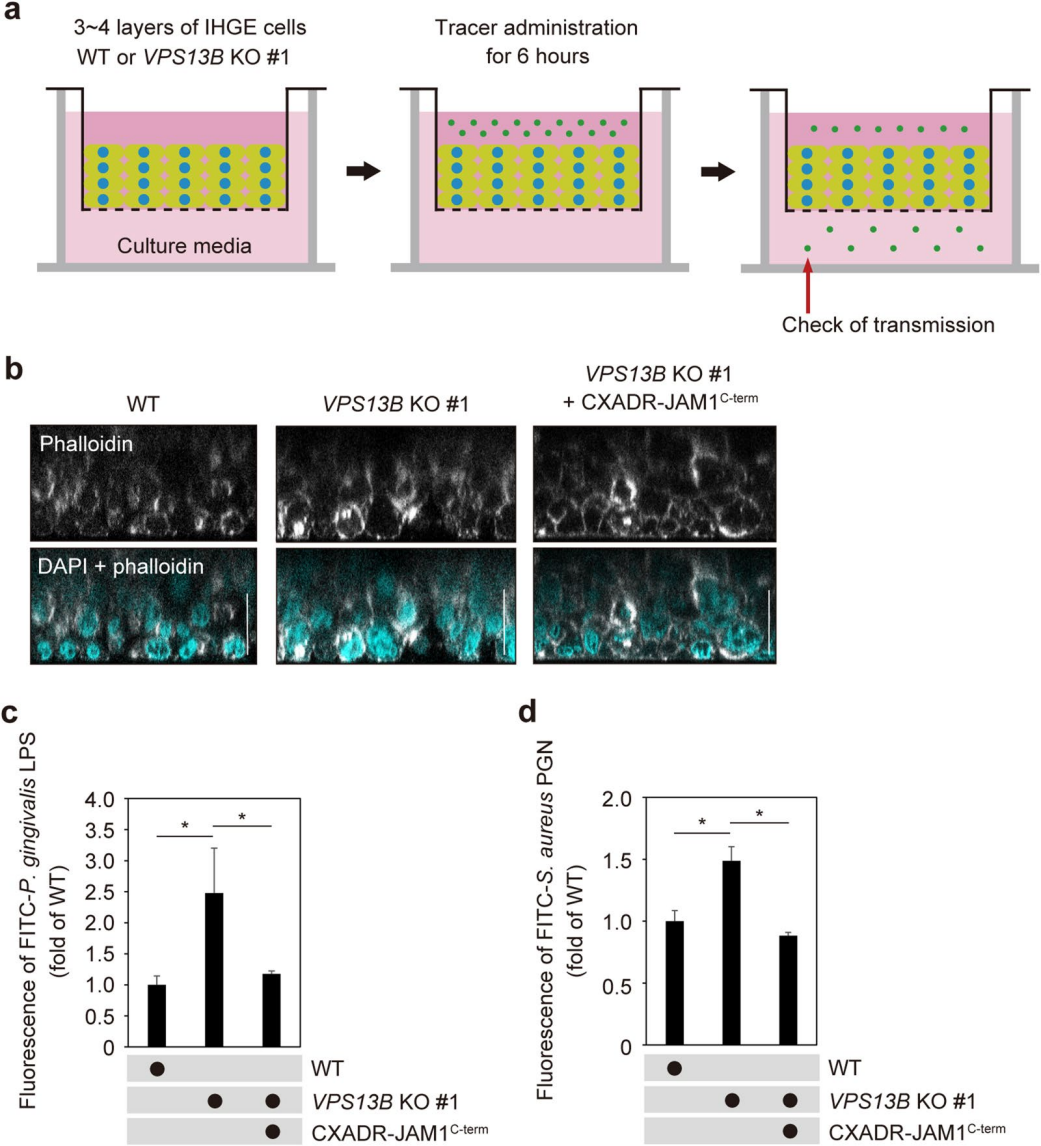
*VPS13B* KO reduces epithelial barrier function of gingival epithelial tissues dependent on extracellular CXADR regions. (**a**) Schematic image of culture-insert system. Gingival epithelial tissue WT or *VPS13B*-KO #1 cells with or without overexpression of HA-inserted CXADR-JAM1^C−term^ were cultured in the upper compartments. FITC-labeled tracers were then added to culture medium in the upper compartments. After 6 h of incubation, transmission of a tracer from the upper to lower compartment was analyzed using spectrometry. (**b**) Gingival epithelial tissue WT or *VPS13B-*KO #1 cells with or without overexpression of HA-inserted CXADR-JAM1^C−term^ on coverslips were fixed, stained with DAPI (cyan) and Alexa Fluor 633-conjugated phalloidin (gray), and analyzed using confocal microscopy. Scale bars, 30 μm. (**c**, **d**) Tissue permeability to *P. gingivalis* LPS (C) or *S. aureus* PGN (**d**). Results are expressed as fold change relative to the control (WT) and shown as the mean ± SD of eight technical replicates. *p < 0.05, two-tailed *t* test (closed-testing procedure). Data shown are representative of two biological replicates.

Finally, 3D-tissue model WT and *VPS13B* KO cells, with and without overexpression of CXADR-JAM1^C−term^, were constructed using a previously reported cell accumulation technique^20,30^ (Fig. 7a). Establishment of gingival epithelial tissues was confirmed using confocal microscopy (Fig. 7b), then those were treated with FITC-labeled *P. gingivalis* LPS or *S. aureus* PGN, and subjected to permeability assays. Six hours following administration, the tissues showed a significant increase in permeability for both FITC-labeled *P. gingivalis* LPS (Fig. 7c) and *S. aureus* PGN (Fig. 7d) associated with *VPS13B* KO, while that was abrogated by overexpression of CXADR-JAM1^C−term^. Cell surface CXADR is thus indicated to be involved in *VPS13B* KO-mediated permeability of gingival epithelium to LPS and PGN.

Based on the results obtained, it was concluded that VPS13B is related to CXADR-dependent barrier function in human gingival epithelium. Furthermore, its abrogation is suggested to be involved in increased permeability of the gingival epithelial barrier in patients affected by Cohen syndrome.

## Discussion

This is the first known report to present findings showing that a tight junction-related protein can be abnormally degraded due to gene mutations, leading to periodontitis-associated disorders. Loss of *VPS13B* was found to result in CXADR being missorted into lysosomes, which leads to its excessive degradation. Notably, this mechanism of endogenous CXADR degradation is different from the consequences of *Porphyromonas gingivalis* infection previously reported^9^.

Greater than 65% of individuals affected by Cohen syndrome experience repeated oral mucosal ulcers^2^. CXADR binding with extracellular matrix proteins such as fibronectin has been observed to occur via IG-LIKE domains^31^, while fibronectin helps wound healing by forming a matrix and interacting with cells. Additionally, Cohen syndrome has been reported to be accompanied by development arrest^2^. Using immunohistochemistry analysis, CXADR expression has been detected in the human central nervous system^32^, while findings showing ubiquitous mRNA expression of *CXADR* in human brain tissues have also been reported^33^. Moreover, patients with Cohen syndrome are affected by fat storage complications and show an increased risk of type 2 diabetes^34^. Beta cells in the pancreas of type 2 diabetes patients become damaged and lose function, leading to an elevated blood sugar level, while the specific isoform of CXADR (CAR SIV) has been found to be expressed in human pancreatic beta cells^35^. Furthermore, Cohen syndrome patients show ocular findings indicating high myopia and corneal ectasia^36^. Generally, keratoconus is a noninflammatory disorder that leads to corneal thinning, protrusion, and corneal ectasia. The epithelial barrier is critical for maintaining the structural integrity and normal functions of the cornea^37^, in which tight junctions play an important role in establishment of corneal homeostasis. It has been shown that human corneal stromal stem cells express *CXADR*^38^. Considering CXADR expression in each tissue and organ, CXADR may be key to understand the pathogenesis of Cohen syndrome-associated findings.

Palmitoylation is a post-translational lipid modification, by which palmitic acid is added via a thioester bond through a thiol group on a cysteine residue of the target protein. The cytoplasmic tail of CXADR, but not JAM1, possesses two palmitoylation sites (Cys259 and Cys260)^39^, which has been reported to be involved in stable expression on the plasma membrane^10^. Palmitoylation is thought to be important for transport of target proteins to lipid microdomains on the plasma membrane, while N-terminal portions of the VPS13 family protein have been found to be involved in lipid transfer between organelle membranes^40^. Thus, VPS13B-mediated lipid logistics in membranes, especially the Golgi apparatus, may be involved in appropriate transport of CXADR to the plasma membrane.

In addition to palmitoylation sites, the C-terminus of CXADR contains the PDZ (PSD-95/Disc-large/ZO-1) domain used for assembly of large protein complexes, cell signaling, or intracellular trafficking. CXADR and PDZ-domain-containing proteins are known to regulate CXADR localization^41^. Furthermore, findings indicating the importance of CXADR within epithelial junctions, where it functions as an adhesion protein interacting with and potentially modulating the trafficking of molecules in the key PDZ domain, have been presented^11,42–44^. Cleavage of proteins in PDZ domains has effects on cell polarity, which induce loss of epithelial integrity^45^. Generally, AMP-activated catalytic subunit alpha 2 (PRKAA2, also known as AMPK) activates the epithelial junction^46^. Activation of AMPK results in interactions with scaffold proteins of the transmembrane, such as zonula occludens-1 (ZO-1), contributing to the early stage of epithelial junction assembly^47,48^. Involvement of phospho-PRKAA2 in the integrity of barrier function of human colon epithelial tissues has also been shown^49^, while phosphorylation of protein kinase AMP-activated catalytic subunit alpha 2 (PRKAA2) was found to be decreased in fibroblasts obtained from Cohen syndrome patients^34^. Thus, VPS13B may be indirectly associated with the phosphorylation cascade in the PDZ domain of tight junction-related proteins, which causes epithelium integrity dysfunction.

RAB6A is ubiquitously expressed and localized in the Golgi apparatus^50^, where it regulates transport of several types of cargo between the Golgi complex and plasma membrane^51^. In experiments with HeLa cells, a human cell line derived from cervical cancer, RAB6A has been shown to be associated with post-Golgi carriers and involved in transport of cargos secreted from exocytosis hotspots. Studies have also found localization of FAM177A1^14^ and SEC23IP in cis-Golgi^15^, and of RAB6A in carriers of the trans-Golgi network to the cell surface^52^. VPS13B is likely a multifunctional protein in the Golgi apparatus and may been related to selective effects noted following knockdown of each gene related to CXADR transport in the present study.

Although periodontal diseases caused by various genetic disorders including Cohen syndrome are associated with neutropenia, *VPS13B-*mutated cases without neutropenia have also been reported^53^. To promote neutrophil differentiation and proliferations, Cohen syndrome patients usually receive treatment with granulocyte colony stimulating factor, which has been shown to have deleterious effects on inflammatory diseases^54^. Thus, further analysis of VPS13B association with CXADR may be important to understand the etiology of periodontitis in Cohen syndrome patients.

## Experimental procedures

### Cell culture

This study was conducted in compliance with the Declaration of Helsinki and all human subjects who participated provided informed consent to the study protocol, which was reviewed and approved by the ethics committee of The Osaka University Graduate School of Dentistry (R2-E8-1). IHGE cells (epi 4, kindly provided by Shinya Murakami, The Osaka University) were maintained in Humedia KG-2 (Kurabo), as previously described^55^. Three-dimensional culturing of IHGE cells was performed as previously described^20,30^, with some modifications. Briefly, IHGE cells collected by centrifugation after trypsinization were alternatively incubated for 3 min with 0.2 mg/mL^− 1^ fibronectin (Sigma-Aldrich) in 0.1 mg/mL^− 1^ gelatin solution (Nacalai tesque). After three immersion steps, single-cell surfaces were coated with fibronectin/collagen nanofilm. For tissue morphological analysis, a total of 2 × 10^6^ cells coated with fibronectin/collagen were seeded onto coverslips coated with a vitronectin solution (A14700, Invitrogen) diluted 1/100 (v/v) in PBS in 24-well plates (Iwaki). After 36 h of incubation, tissues were fixed and subjected to experiments, with the results analyzed with use of a confocal microscope (TCS SP8; Leica Microsystems). For permeability experiments, a total of 1 × 10^6^ cells with fibronectin/collagen were seeded into 24-well cell culture inserts (353096, Corning).

### Antibodies, plasmids, and reagents

Antibodies, plasmids, and reagents used in this study are shown in Supplementary Table S1.

### Plasmid construction

Plasmids used in this study are shown in Supplementary Table S1. A plasmid encoding HA-inserted CXADR-JAM1^C−term^ and JAM1-CXADR^C−term^ was constructed by placing overlapping fusion PCR from CXADR and JAM1 cDNA of IHGE cells into the pCMV-HA plasmid (Clontech), with KpnI and NotI sites exogenously added. To produce pBApo-EF1α NEO HA-inserted CXADR-JAM1^C−term^ and JAM1-CXADR^C−term^ for establishment of stable cell lines, DNA was subcloned from pCMV and inserted into pBApo-EF1α NEO (Takara) using BamHI and NotI sites. All PCR products and mutations were confirmed by sequencing (FASMAC). Transfection was performed using FuGENE 6 Transfection Reagent (Promega).

### Immunoblotting and immunocytochemistry

Immunoblotting and immunocytochemistry were performed as previously described^20,56^. Briefly, detection of immunoreactive bands was performed using Pierce ELC Western Blotting Substrate (Thermo Scientific) and ChemiDoc XRS (Bio Rad), then images were acquired using the Quantify One software package (Bio-Rad). Confocal microscopic images were acquired with a confocal laser microscope (TCS SP8; Leica Microsystems) using a 64× oil-immersion object lens with a numerical aperture of 1.4 and analyzed using the Application Suite X software package (Leica Microsystems). The degree of colocalization was quantified using Manders’ overlap coefficient calculated with ImageJ (FIJI) software (https://imagej.net/imagej-wiki-static/Colocalization_Analysis) of ten pictures per condition. The Manders’ coefficients between the two channels of each fluorescent image were calculated using the Colocalization Threshold plugin in ImageJ. Three Golgi morphologies (intact, partial and full fragmentation) in Supplementary Fig. 3a were defined, as previously described^57^.

### RNA interference

Plasmid encoding shRNA was constructed by ligation of linear DNA (Sigma-Aldrich) into pSIREN-RetroQ (Clontech). Target sequences for shRNA used for generating the siRNA duplex are shown in Supplementary Table S2. The pSIREN-RetroQ-shLuc plasmid was produced as previously described^20,25^. IHGE cells were transfected with shRNA-encoding plasmid using FuGENE 6 (Promega). Seventy-two hours after transfection, cells stably expressing shRNA were selected with puromycin (2 µg mL^− 1^).

### Quantitative real-time PCR

Reverse transcription reactions were performed using ReverTra Ace qPCR RT Master Mix (Toyobo). Go-Taq (Promega) was used for executing PCR. Quantitative real-time PCR was performed as previously described^20^. Briefly, total RNA was extracted from IHGE cells using an RNeasy Micro kit (Qiagen). Complementary DNA was then synthesized using ReverTra Ace qPCR RT Master Mix (Toyobo) and real-time PCR was performed using a Rotor Gene Q (Qiagen) with THUNDERBIRD SYBR qPCR Mix (Toyobo). Primer sequences are shown in Supplementary Table S3. The amplicon level in each sample was normalized based on the corresponding level of *β*-*ACTIN* mRNA content using the 2^−ΔΔCt^ method.

### Establishment of IHGE cells with *VPS13B* KO

A CRISPR/Cas9 Genome Knockout Kit, designed to target the human gene *VPS13B* (KN412550), was purchased from Origene. The target sequence was human *VPS13B #1* (5’-AGTGAAAGCTGTAGATCCGA-3’, in exon 1) and #2 (5’- CGAGTTAAAGTTGGATGTGC-3’, in exon 1). This guide RNA sequence was designed to insert a puromycin-resistant gene along with a termination codon in the exon 1 region of the gene. IHGE cells were transfected using FuGENE6 (Promega) with the guide vector and linear donor. At 72 h after transfection, knockout cells were selected with puromycin (2 µg mL^− 1^; InvivoGen), then knockout was evaluated by immunofluorescence staining.

To establish IHGE cells, *VPS13B* KO cells stably expressing HA-inserted CXADR-JAM1^C−term^ and JAM1-CXADR^C−term^ were generated, then transfected with pBApo-EF1α NEO HA-inserted CXADR-JAM1^C−term^ or JAM1-CXADR^C−term^ plasmid using FuGENE6 (Promega). After 72 h of incubation, cells were selected using G418 (200 µg mL^− 1^; InvivoGen).

### Isolation of membrane and cytosolic fractions

Plasma membrane isolation was performed as previously described^22,58^. Briefly, IHGE cells in a cell culture plate (100 mm, 3020 − 100, IWAKI) were washed with PBS and suspended in 10 mL of ice-cold distilled water for 1 h at 4℃ to rupture the cells. The plates were then washed with PBS to remove intracellular organelles, and plasma membrane attached to the plates was scraped and lysed in 250 µL of lysis buffer (2 M thiourea, 7 M urea, 3% CHAPS, 1% Triton X-100) on ice for 30 min. Next, centrifugation at 17,400 x *g* was performed for 30 min at 4℃ and the supernatant used as the plasma membrane fraction.

### Epithelial barrier functional assay

FITC-tracers were prepared as previously described^20^. To assess the barrier function of cell layers and tissues, in vitro epithelial permeability assays were performed using 12-well (353180; Corning) and 24-well (353096, Corning) cell culture inserts, respectively. Fluorescence intensity was determined with use of a 1420 ARVO X reader (PerkinElmer). Data were analyzed using the WorkOut Plus software package (PerkinElmer).

### Statistical analysis

P values were determined using a two-tailed *t* test or one-tailed Dunnett’s test with the Excel software package (Microsoft), with *p* < 0.05 considered to indicate significance.

## Supplementary Information

Below is the link to the electronic supplementary material.

Supplementary Material 1

## Data Availability

Data supporting the findings of the present study are available from the corresponding author upon reasonable request.
