# Supplementary material for: VPS13B, gene responsible for Cohen syndrome, regulates gingival epithelial barrier function via intracellular trafficking of coxsackievirus and adenovirus receptor
Source: Sci Rep. 2026 Feb 24;16:10313. doi: 10.1038/s41598-026-40840-9 (PMC13032043; doi:10.1038/s41598-026-40840-9)

VPS13B, gene responsible for Cohen syndrome, regulates gingival epithelial barrier function via intracellular trafficking of coxsackievirus and adenovirus receptor

Risako Matsumura[1, 5], Keita Tanigaki[2], Naoko Sasaki[3], Tsukasa Tamamori[1], Shunsuke Yamaga[2], Akito Sakanaka[1], Atsuo Amano[1], Michiya Matsusaki[4], Hiroki Takeuchi[2, 5], Masae Kuboniwa[1]

[1]*Department of Preventive Dentistry, Graduate School of Dentistry, The Osaka University, Suita-Osaka 565-0871, Japan*

[2]*Department of Preventive Dentistry, The Osaka University Dental Hospital, Suita-Osaka 565-0871, Japan*

[3]*Joint Research Laboratory (TOPPAN) for Advanced Cell Regulatory Chemistry, Graduate School of Engineering, The Osaka University, Suita-Osaka 565-0871, Japan*

[4]*Department of Applied Chemistry, Graduate School of Engineering, The Osaka University, Suita-Osaka 565-0871, Japan*

[5]*These authors contributed equally to this work.*

Supplementary Table S1. Antibodies, plasmids, and reagents.

Material	Source	Cat. No.
Mouse monoclonal anti-HA	MBL	M180-3
Rabbit monoclonal anti-HA	Cell Signaling Technology	3724
Mouse monoclonal anti-JAM1	Sigma-Aldrich	SAB4200468
Rabbit monoclonal anti-CXADR	Sino Biological	10799-R271
Rabbit polyclonal anti-VPS13B	Proteintech	24505-1-AP
Mouse monoclonal anti-β-ACTIN	Sigma-Aldrich	M177-3
Mouse monoclonal anti-GM130	BD	610823
Mouse monoclonal anti-p230 trans Golgi (GOLGA4)	BD	611280
Mouse monoclonal anti-LAMP1	Santa Cruz	sc-20011
FITC-conjugated goat anti-mouse IgG	MBL	A-11001
Alexa Fluor 488-conjugated goat anti-mouse IgG	Abcam	ab150113
Alexa Fluor 488-conjugated goat anti-rabbit IgG	Abcam	ab150077
Alexa Fluor 555-conjugated goat anti-mouse IgG	Invitrogen	A21422
Alexa Fluor 555-conjugated goat anti-rabbit IgG	Abcam	ab150078
Alexa Fluor 647-conjugated goat anti-mouse IgG	Cell Signaling Technology	4410
Alexa Fluor 647-conjugated goat anti-rabbit IgG	Invitrogen	ab150079
Alexa Fluor 633 phalloidin	Thermo Fisher Scientific	A22284
Goat anti-mouse antibody conjugated to horseradish peroxidase	Cell Signaling Technology	7076
Goat anti-rabbit antibody conjugated to horseradish peroxidase	Cell Signaling Technology	7074
pSIREN-RetroQ	Clontech	631526
pSIREN-RetroQ-shLuc	Takeuchi *et al*., 2019	-
pBApo-EF1α NEO	Takara	3243
pCMV-HA-inserted CXADR	Takeuchi *et al*., 2021	-
pIRES-Puro-HA-inserted CXADR	Takeuchi *et al*., 2021	-
Bafilomycin A1	Sigma-Aldrich	B1793
EACC	MedChemExpress	HY129111
Fluorescein Labeling Kit-NH2	Dojindo	LK-01
FITC-*P. gingivalis* LPS	Takeuchi *et al*., 2019	-
FITC-*S. aureus* PGN	Takeuchi *et al*., 2019	-
KOD plus Neo	Toyobo	KOD-401
T4 DNA ligase	New England Biolabs	M0202

Supplementary Table S2. shRNA sequences.

shRNA	Sequences (5' to 3')
shVPS13B #1458	GAGTACGAAAGGTTTCACATACC
shRAB6A #263	TGGCAGTTGTTGTTTATGATATC
shRAB6A #549	AAGCAGAGAAGATATGATTGACA
shFAM177A1 #186	TGGTGAAACAATGGAAGAATATA
shSEC23IP #880	TTGAATCTTGAAGAAATCTATAA
shSEC23IP #2941	CTGTTACTACTTAAAGAAATTTA
shSTX13 #526	CAGGATTTGGAACTTATTAAAGA
shSTX13 #587	TGGATGTCAATCAGATATTTAAA

Supplementary Table S3. Primers.

Primers	Sequences (5' to 3')	Anealing temperature, time (cycles)
β-ACTIN forward	GCATGGGTCAGAAGGATTCCT	58°C, 15 seconds (45)
β-ACTIN reverse	TCGTCCCAGTTGGTGACGAT	58°C, 15 seconds (45)
VPS13B forward	AGGCACAGCTCCTCCAGATA	58°C, 15 seconds (45)
VPS13B reverse	AACACGTCCACTGGTTCCTC	58°C, 15 seconds (45)
CXADR forward	CAGTGCCTGTTGCGTCTAAA	58°C, 15 seconds (45)
CXADR reverse	CTTTGGAGGTGGCACATCTT	58°C, 15 seconds (45)
RAB6A forward	GAGCAAAGCGTTGGAAAGAC	58°C, 15 seconds (45)
RAB6A reverse	TGACCTGCTGTGTCCCATAA	58°C, 15 seconds (45)
FAM177A1 forward	TGTGGAACGAGGAGAAGCCGTC	58°C, 15 seconds (45)
FAM177A1 reverse	CCAGTTCTACATTTTCAAAGCCTC	58°C, 15 seconds (45)
SEC23IP forward	GAACCCAGACTTCAAAGGAGGTG	58°C, 15 seconds (45)
SEC23IP reverse	AGCAAGAGGTCCAGGGCACTTT	58°C, 15 seconds (45)
STX13 forward	GCCAATGTGGAAAGCTCAGAGG	58°C, 15 seconds (45)
STX13 reverse	TCACTGACAGGACAAGCACCAG	58°C, 15 seconds (45)

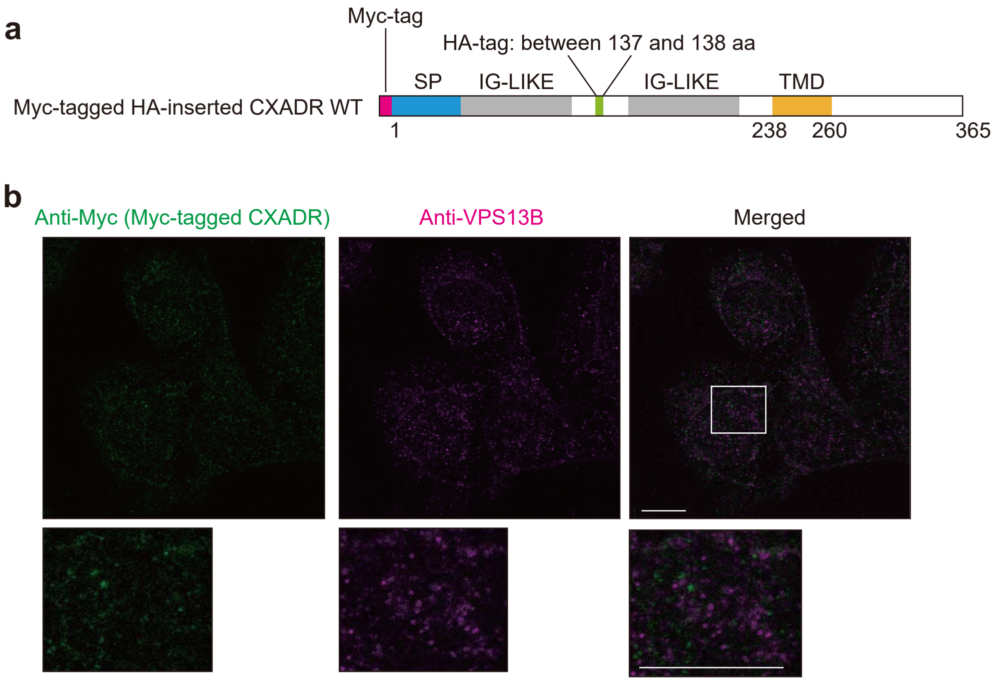

Supplementary Figure 1. Confocal microscopic analysis of VPS13B and Myc-tagged HA-inserted CXADR in IHGE cells

(a) Schematic view of Myc-tagged HA-inserted CXADR. SP (cyan), IG-LIKE (gray), and TMD (yellow) domains are indicated. Myc-tag or HA-tag is shown in magenta or green, respectively. **(b, c)** IHGE cells stably expressing Myc-tagged HA-inserted CXADR were fixed, then stained with rabbit polyclonal anti-VPS13B (magenta: Alexa Fluor 555) and either of mouse monoclonal anti-Myc (green in b: Alexa Fluor 488) or mouse monoclonal anti-HA (green in c: Alexa Fluor 488), respectively. Cells were then analyzed using confocal microscopy. Scale bars, 10 µm. Result is representative of ten technical replicates. **(d)** Comparison of co-efficiency between VPS13B and either of Myc-tagged or HA-inserted CXADR, with VPS13B as the denominator in b) and c). Values are shown as the mean ±SD of ten technical replicates. *$p<0.05$, two-tailed *t* test. Data shown are representative of two biological replicates.

(Supplementary Figure 1)

c

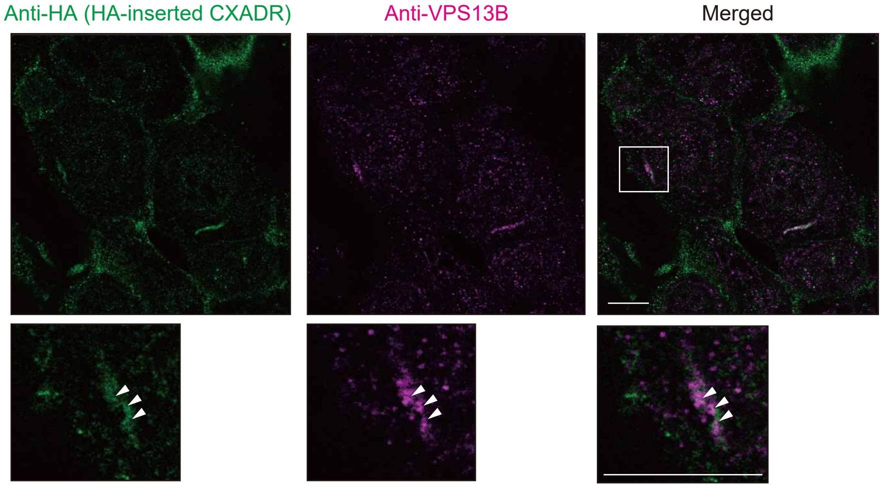

d

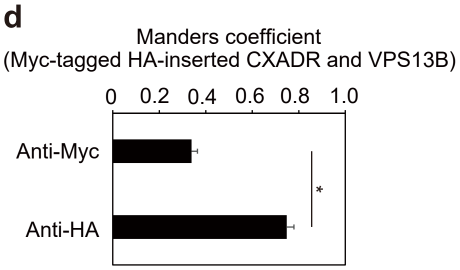

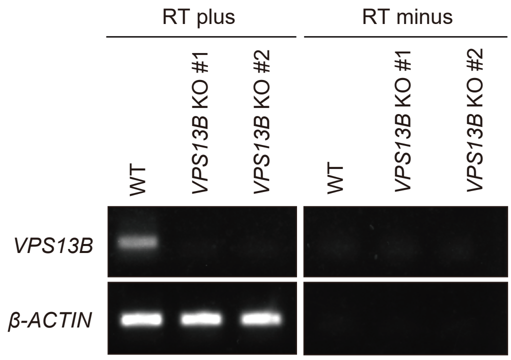

Supplementary Figure 2. RT-PCR analysis of *VPS13B* gene in *VPS13B*-KO IHGE cells.

β-ACTIN used as control. RT, reverse transcription.

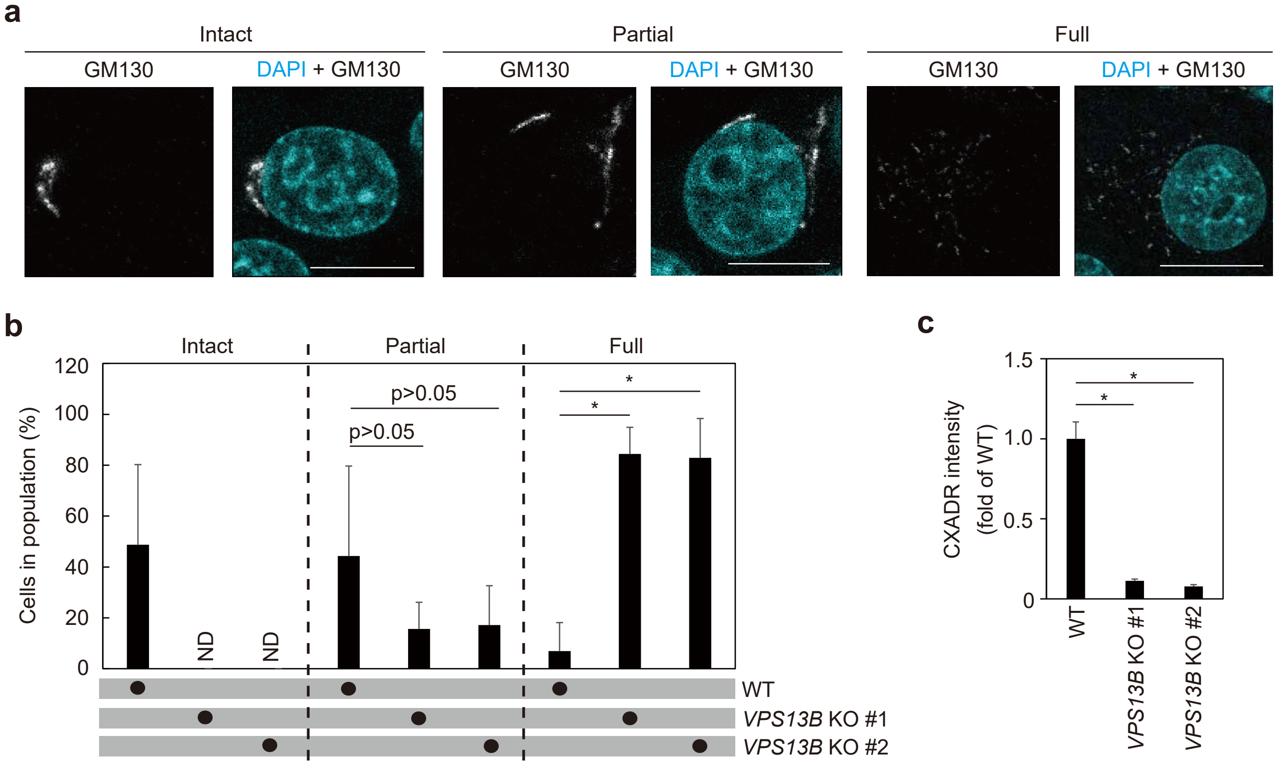

Supplementary Figure 3. Confocal microscopic analysis related to Figure 2b and 2c.

(a) Representative images of the three possible morphologies of the Golgi (gray, GM130) that defined as intact, partially and fully fragmented Golgi in Figure 2b. Result is representative of ten biological replicates. Bars, 10 μm. **(b)** Comparison of Golgi morphologies between IHGE WT or *VPS13B*-KO cells in a). Values are shown as the mean ±SD of seven technical replicates. *$p<0.05$, one-tailed Dunnett's test. **(c)** Comparison of CXADR fluorescent intensity between IHGE WT or *VPS13B*-KO cells in Figure 2c. Values are shown as the mean ±SD of ten technical replicates. *$p<0.05$, one-tailed Dunnett's test. ND, not detected. Data shown are representative of two biological replicates.

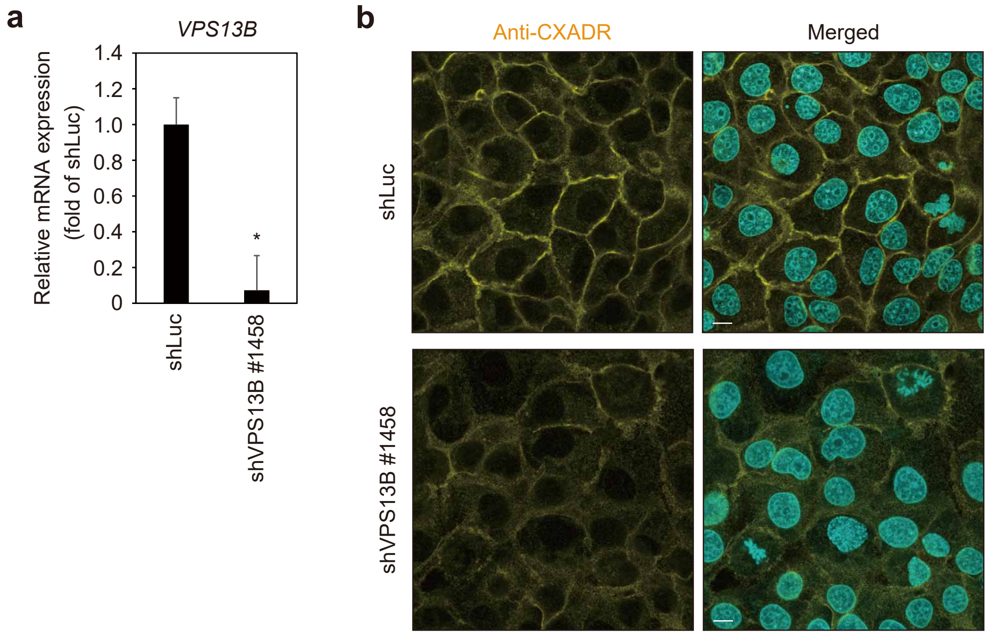

Supplementary Figure 4. *VPS13B* knockdown decreases cell surface localization of CXADR in IHGE cells.

(a) *VPS13B* mRNA expressions in IHGE cells stably expressing shLuc or shVPS13B #1458 are shown as fold change relative to shLuc-expressing cells, with the mean value ±SD of five technical replicates presented. Significant differences were determined using a two-tailed *t* test (*p<0.05). Data shown are representative of two biological replicates. **(b)** IHGE cells expressing shLuc or shVPS13B #1458 were fixed, then stained with DAPI (cyan) and rabbit monoclonal anti-CXADR (yellow: Alexa Fluor 555), and analyzed by confocal microscopy. Scale bars, 10 μm. Result is representative of two biological replicates.

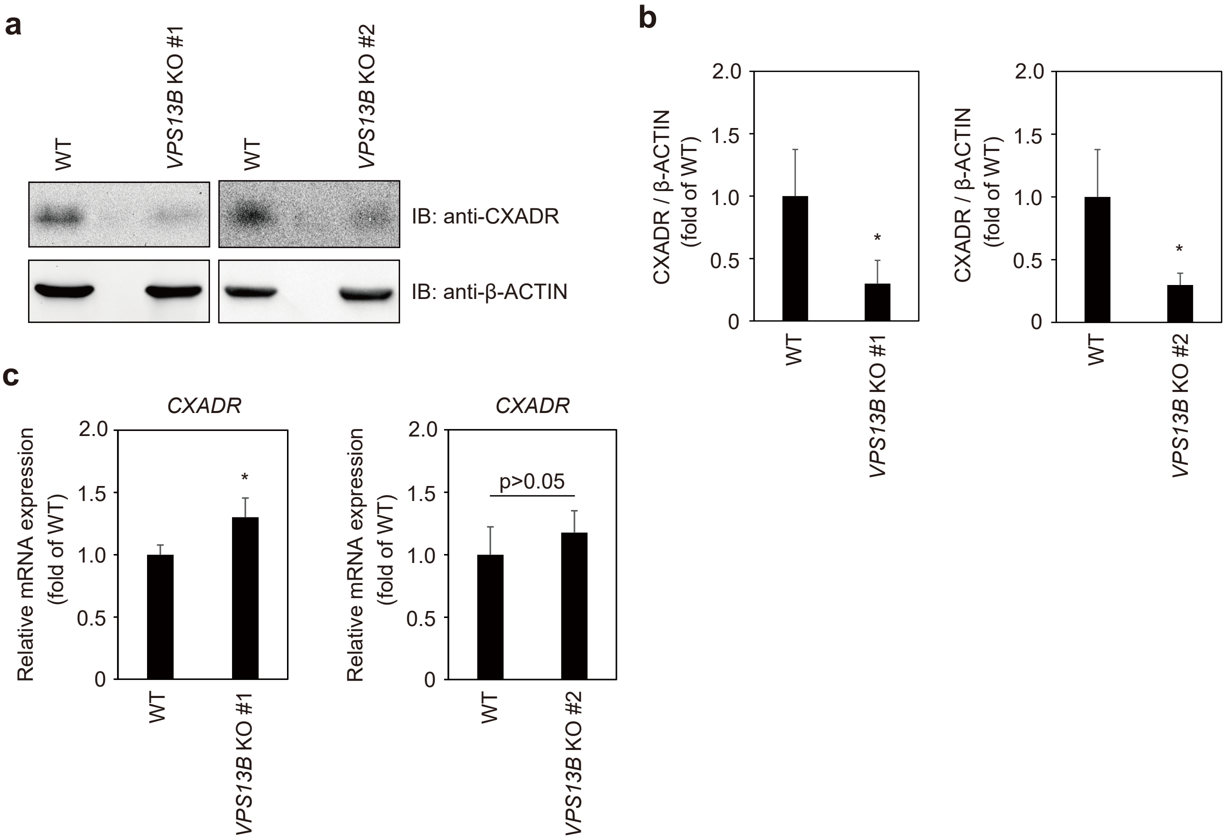

Supplementary Figure 5. Protein and gene levels of CXADR in WT IHGE or *VPS13B*-KO cells.

(a) CXADR expression in IHGE WT and *VPS13B*-KO (left #1, right #2) cells was analyzed by immunoblotting with the indicated antibodies. β-ACTIN was used as a loading control. IB, immunoblot. Result is representative of three biological replicates. **(b)** Densitometry of CXADR in a) is expressed as fold change and vales are shown as the mean ±SD of three biological replicates. *$p<0.05$, two-tailed *t* test. Result is representative of two biological replicates. **(c)** Relative mRNA expression of *CXADR* in IHGE WT and *VPS13B* KO (left #1, right #2) is expressed as fold change relative to *β-ACTIN* expression and values are shown as the mean ±SD of five technical replicates. *$p<0.05$, two-tailed *t* test. Data shown are representative of two biological replicates.

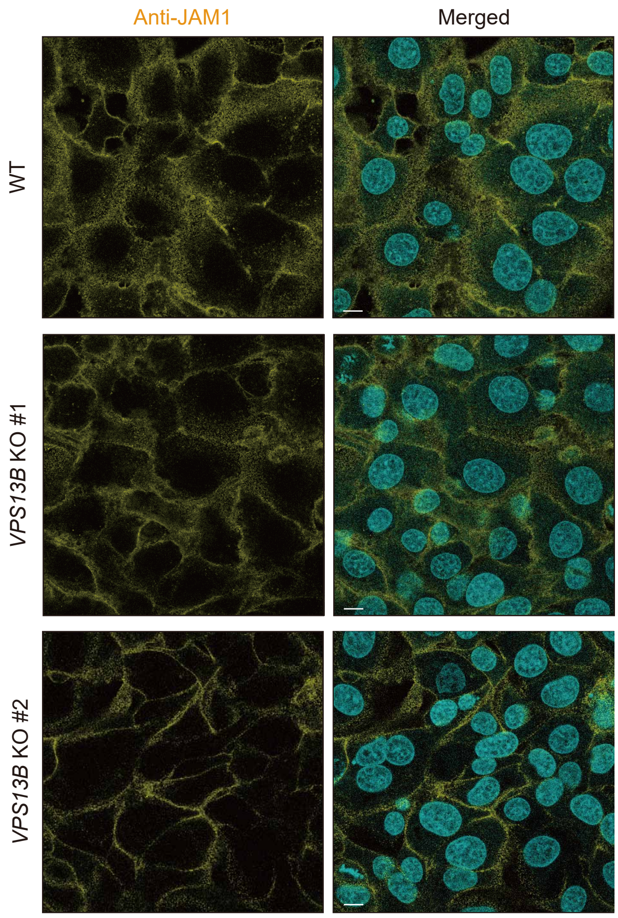

Supplementary Figure 6. Effects of *VPS13B* KO on JAM1 localization in IHGE cells. IHGE WT and *VPS13B*-KO (#1, #2) cells were fixed, then stained with DAPI (cyan) and mouse monoclonal anti-JAM1 (yellow: Alexa Fluor 555), and analyzed using confocal microscopy. Scale bars, 10 µm. Result is representative of two biological replicates.

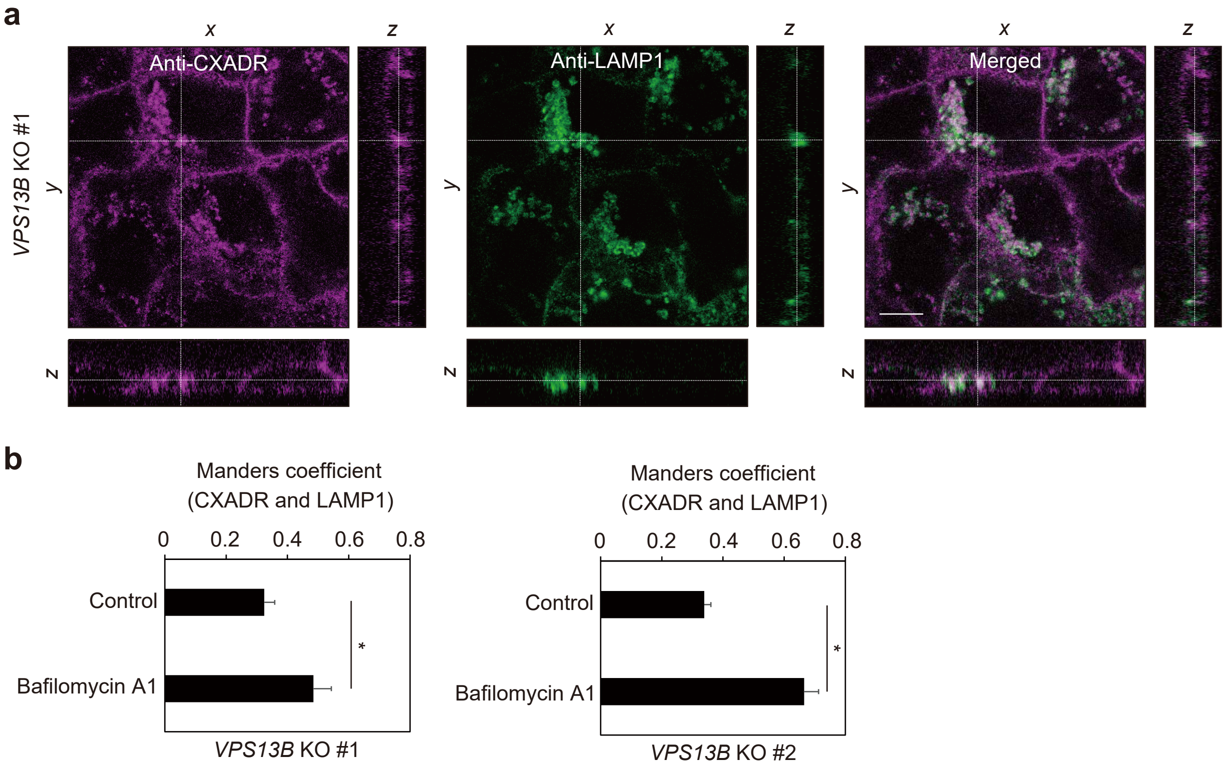

Supplementary Figure 7. Effects of bafilomycin A1 on co-localization of CXADR and LAMP1 in IHGE WT or *VPS13B*-KO cells shown in Figure 3.

(a) IHGE *VPS13B*-KO #1 cells were treated with bafilomycin A1 (100 nM) for 24 hours, then fixed, stained with anti-CXADR (magenta: Alexa Fluor 647) and anti-LAMP1 (green: Alexa Fluor 555), and analyzed using confocal microscopy. Scale bars, 10 μm. **(b)** Comparison of co-efficiency between CXADR and LAMP1, with CXADR as the denominator in Figure 3. Values are shown as the mean ±SD of ten technical replicates. *p<0.05, two-tailed *t* test. Data shown are representative of two biological replicates.

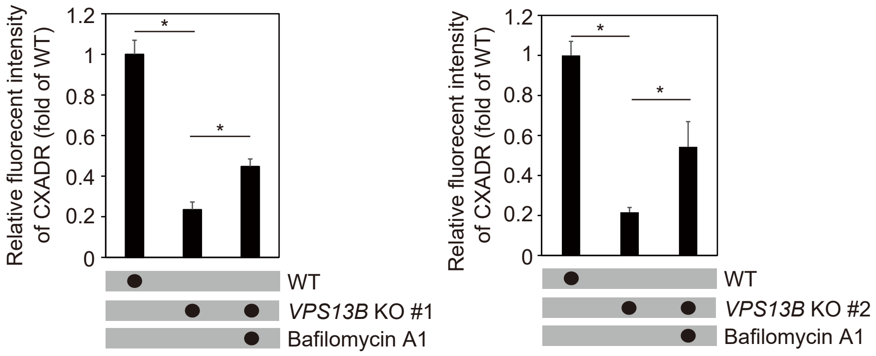

Supplementary Figure 8. Effects of bafilomycin A1 on cell-surface CXADR in IHGE WT or *VPS13B*-KO cells shown in Figure 3.

Comparison of CXADR fluorescent intensity in the indicated cells in Figure 3. Values are shown as the mean ±SD of ten technical replicates. *p<0.05, two-tailed *t* test (closed-testing procedure).

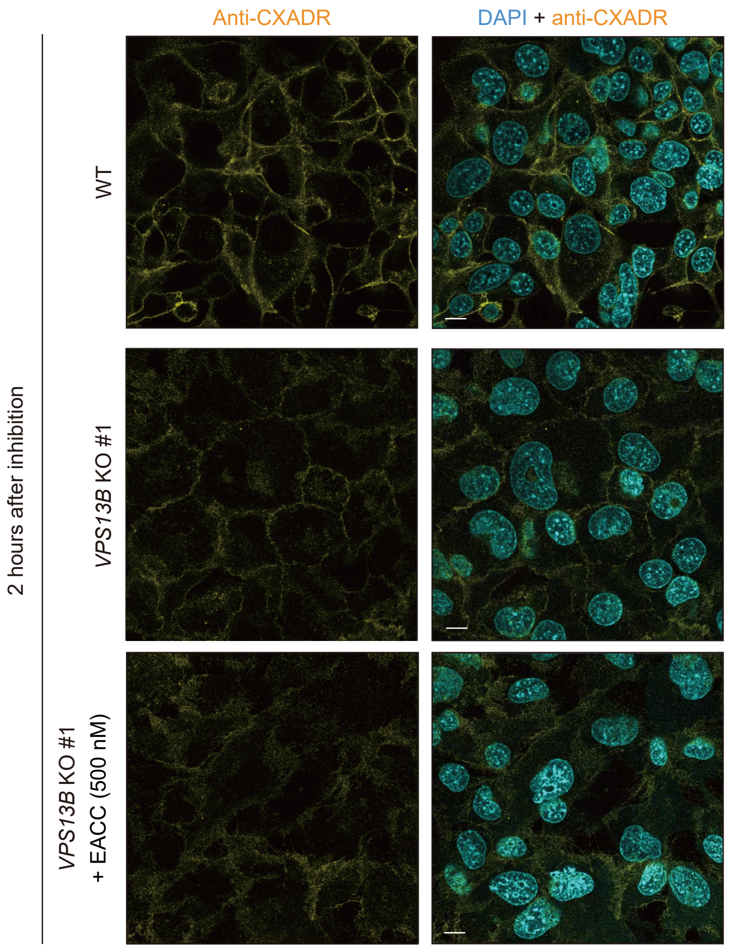

Supplementary Figure 9. CXADR localization in *VPS13B*-KO IHGE cells treated with EACC.

IHGE WT, *VPS13B*-KO #1, and *VPS13B*-KO #2 cells were treated with or without EACC (500 nM) for 2 hours, then fixed, stained with DAPI (cyan) and rabbit monoclonal anti-CXADR (yellow: Alexa Fluor 555), and analyzed using confocal microscopy. Scale bars, 10 µm. Result is representative of two biological replicates.

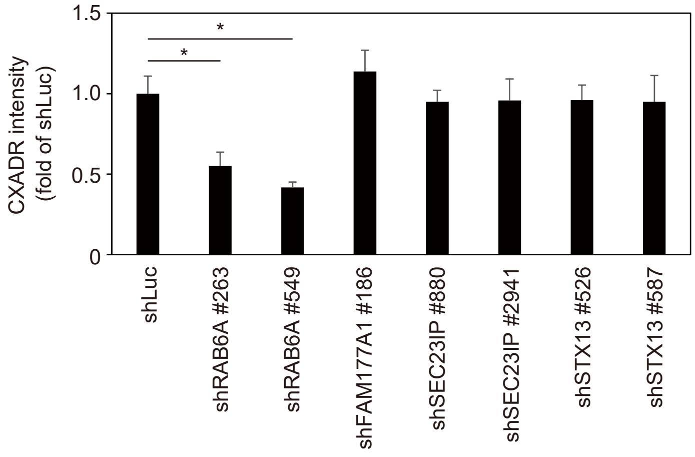

Supplementary Figure 10. Relative CXADR expression in Figure 4c.

Densitometry of confocal microscopic images in Figure 4c. Relative CXADR expression is expressed as fold change and values are shown as the mean ±SD of ten technical replicates. *$p<0.05$, one-tailed Dunnett's test. Data shown are representative of two biological replicates.

a

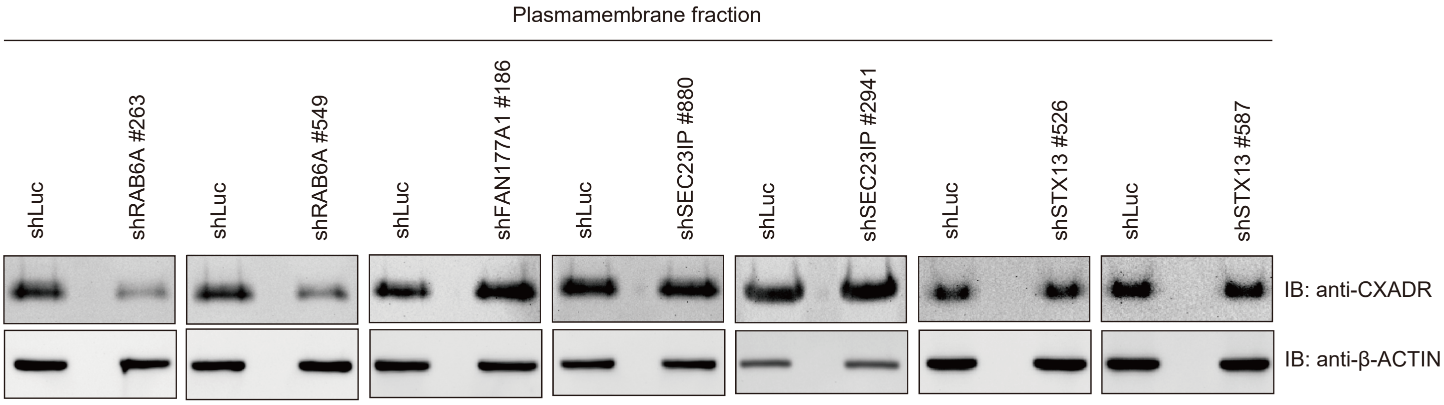

Supplementary Figure 11. Immunoblot analysis of plasma-membrane fractions of IHGE cells expressing shRAB6A, shFAM177A1, shSEC23IP or shSTX13.

(a) Plasma membrane fractions of IHGE cells expressing the indicated shRNA were prepared and immunoblotted with the indicated antibodies. β-ACTIN was used as a loading control. Result is representative of three biological replicates. **(b)** Densitometry of immunoblot in a). Relative CXADR expression is expressed as fold change relative to *β-ACTIN* expression and values are shown as the mean ±SD of three biological replicates. *p<0.05, two-tailed *t* test.

(Supplementary Figure 11)

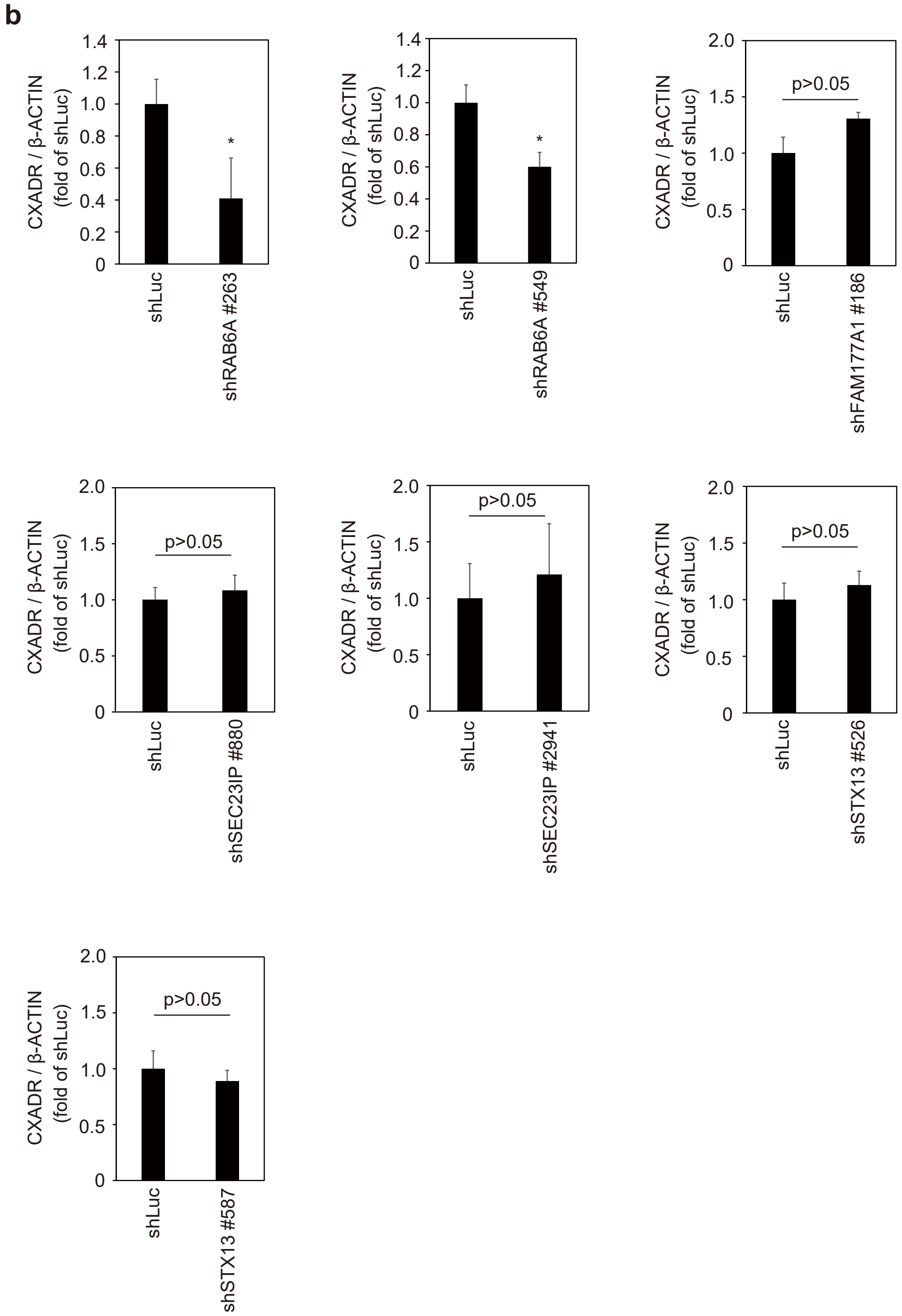

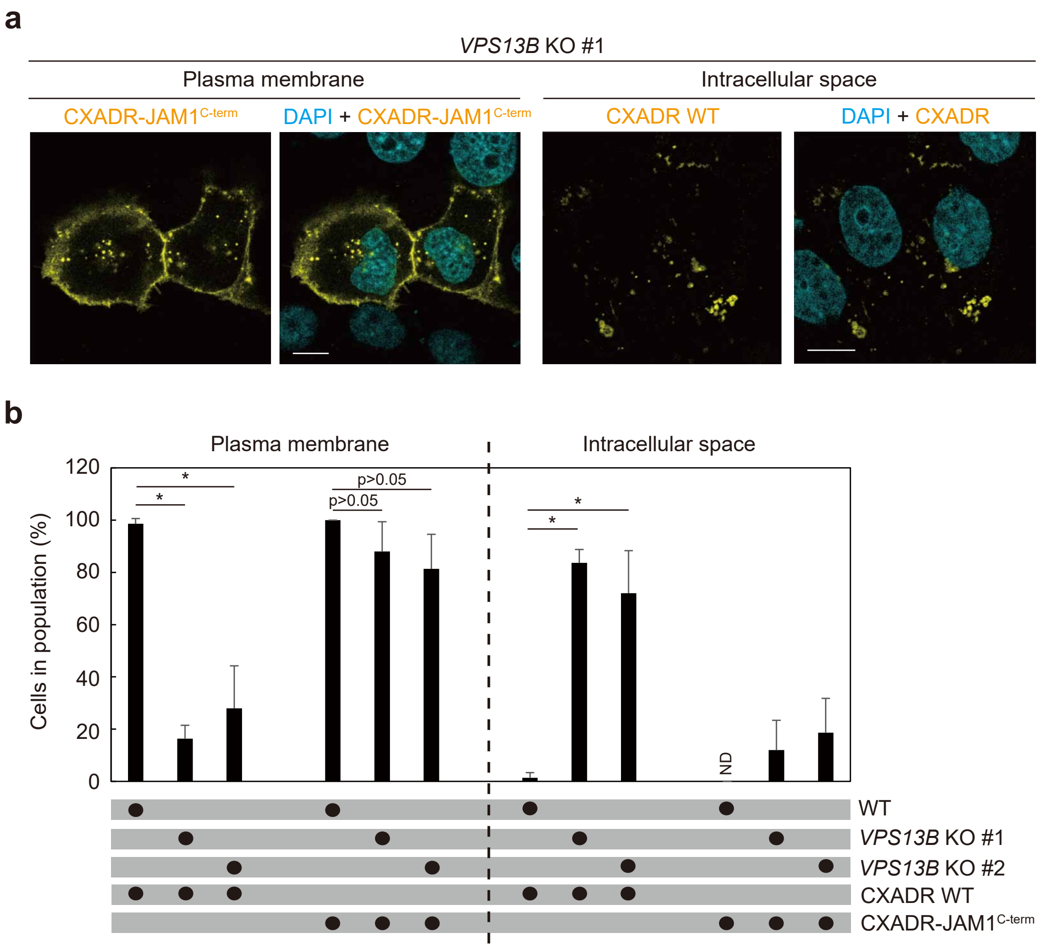

Supplementary Figure 12. Confocal microscopic analysis related to Figure 5b.

(a) Representative images of IHGE cells expressing HA-inserted CXADR (yellow) transported to the plasma membrane (left, HA-inserted CXADR-JAM1$^{C\text{-term}}$ in *VPS13B*-KO cells) and retained in intracellular space (right, HA-inserted CXADR WT in *VPS13B*-KO cells) in Figure 5b. Result is representative of ten technical replicates. Bars, 10 µm. **(b)** Comparison of CXADR localization in IHGE WT or *VPS13B*-KO cells in Figure 5b. Values are shown as the mean ±SD of ten technical replicates. *$p<0.05$, one-tailed Dunnett's test. ND, not detected. Data shown are representative of two biological replicates.

a

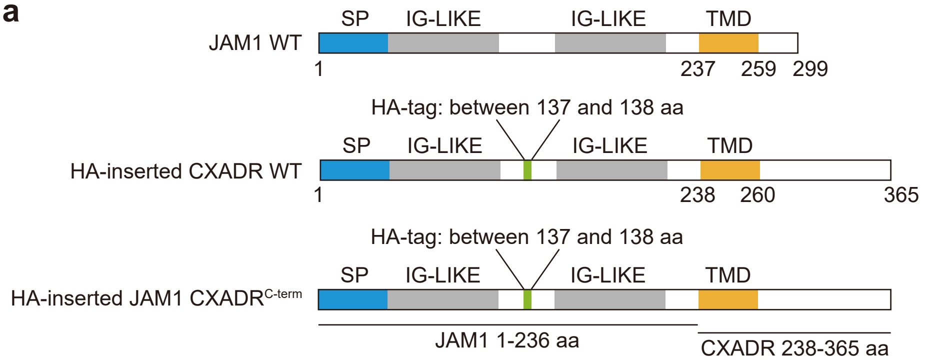

Supplementary Figure 13. JAM1 WT and CXADR$^{C\text{-}term}$ localization in IHGE *VPS13B*-KO cells.

(a) Schematic view of JAM1 WT, HA-inserted CXADR WT, and HA-inserted JAM1-CXADR$^{C\text{-}term}$ cells. SP (cyan), IG-LIKE (gray), and TMD (yellow) domains are indicated. HA-tag is shown in green. **(b)** IHGE WT and *VPS13B*-KO (#1, #2) cells transiently expressing HA-inserted JAM1 WT or JAM1-CXADR$^{C\text{-}term}$ were fixed, then stained with DAPI (cyan) and rabbit monoclonal anti-HA (yellow: Alexa Fluor 555), and analyzed using confocal microscopy. Scale bars, 10 µm. Result is representative of two biological replicates.

(Supplementary Figure 13)

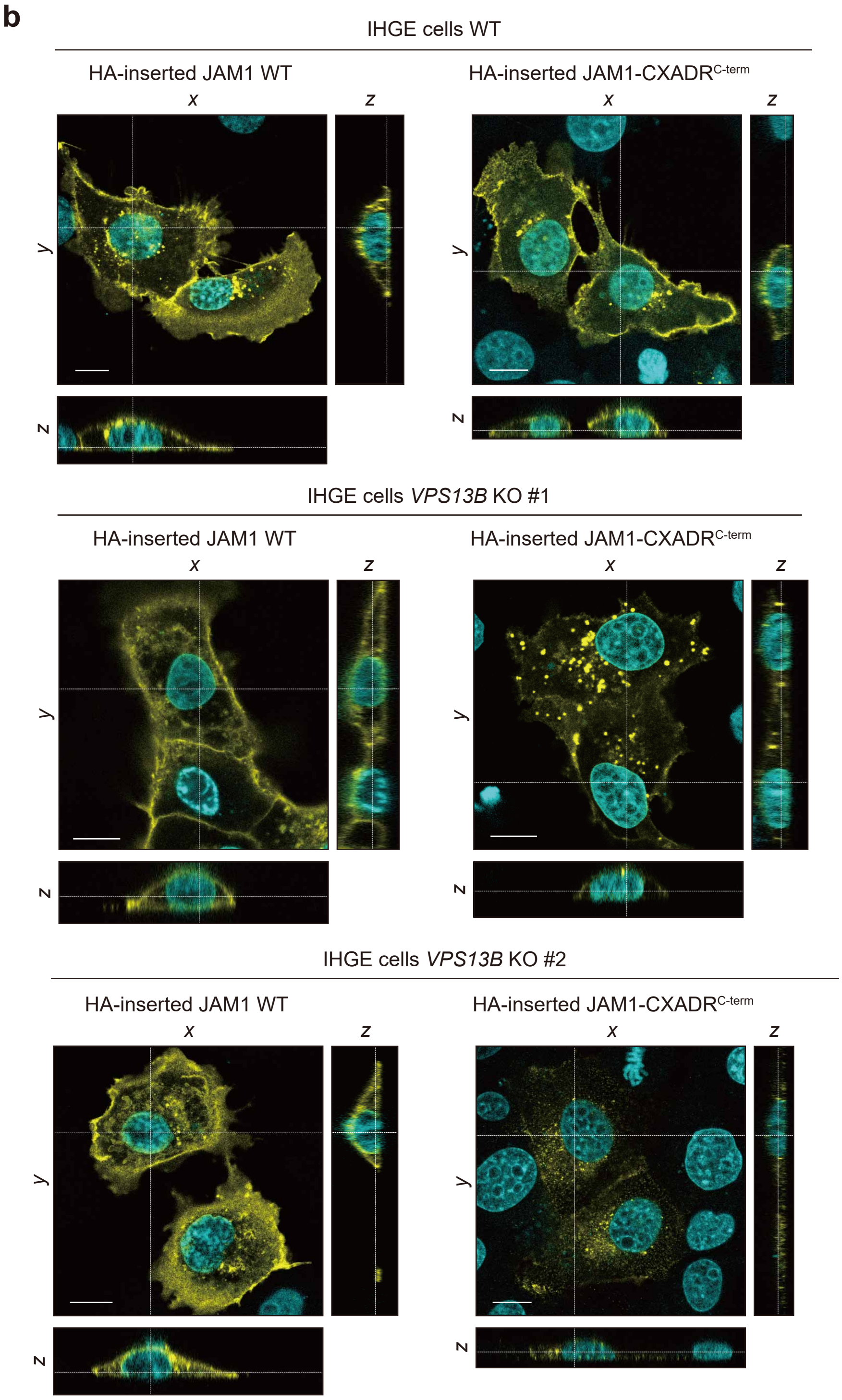

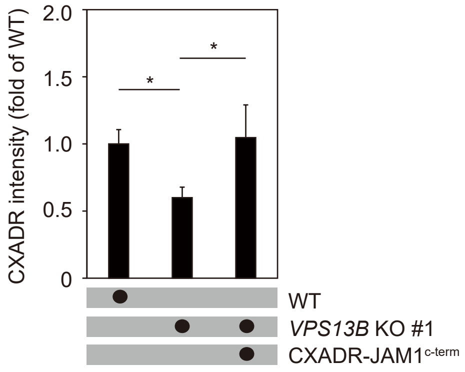

Supplementary Figure 14. Relative CXADR expression in Figure 6b.

Densitometry of confocal microscopic images in Figure 6b. Relative CXADR expression is expressed as fold change and values are shown as the mean ±SD of ten technical replicates. *p<0.05, two-tailed *t* test (closed-testing procedure). Data shown are representative of two biological replicates.

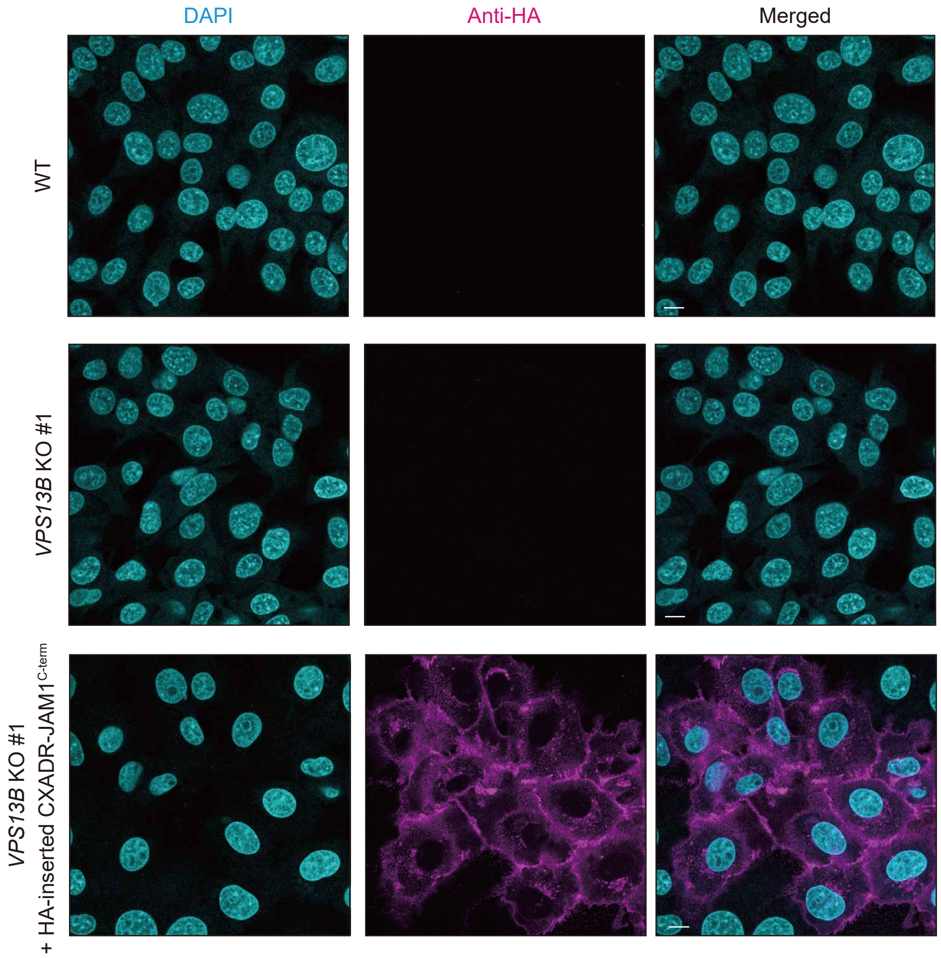

Supplementary Figure 15. Confocal microscopic images of IHGE WT or *VPS13B*-KO cell layer with or without CXADR-JAM1$^{\text{C-term}}$ overexpression.

IHGE WT and *VPS13B*-KO #1 cell layers with or without overexpression of CXADR-JAM1$^{\text{C-term}}$ on coverslips were fixed, then stained with DAPI (cyan) and anti-HA (magenta: Alexa Fluor 647), and analyzed using confocal microscopy. Scale bars, 10 µm. Result is representative of two biological replicates.

Supplementary Figure 16. Immunoblotting performed in this study.

Supplementary Figure 2

PCR: *VPS13B*

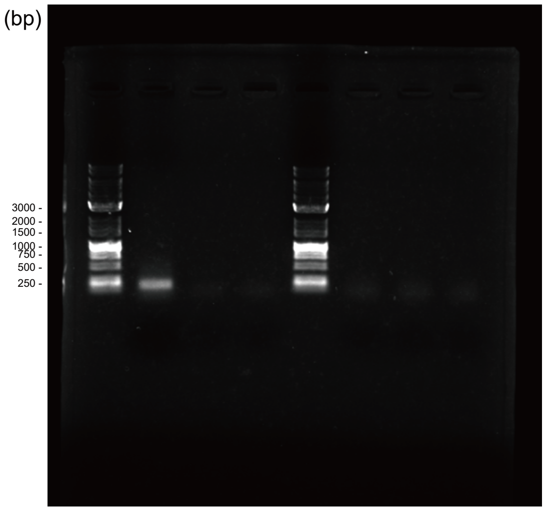

Uncropped image

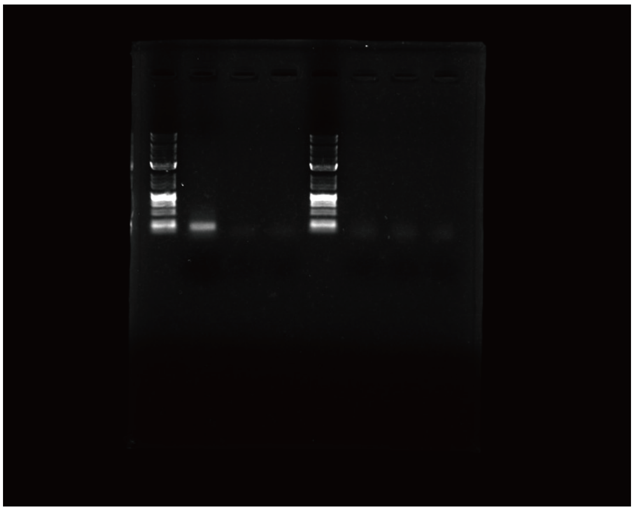

PCR: *β-ACTIN*

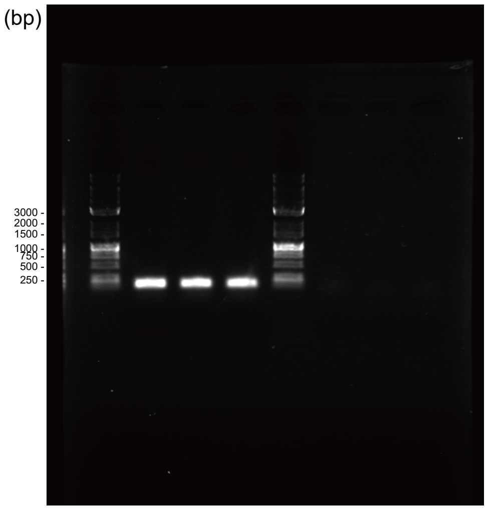

Uncropped image

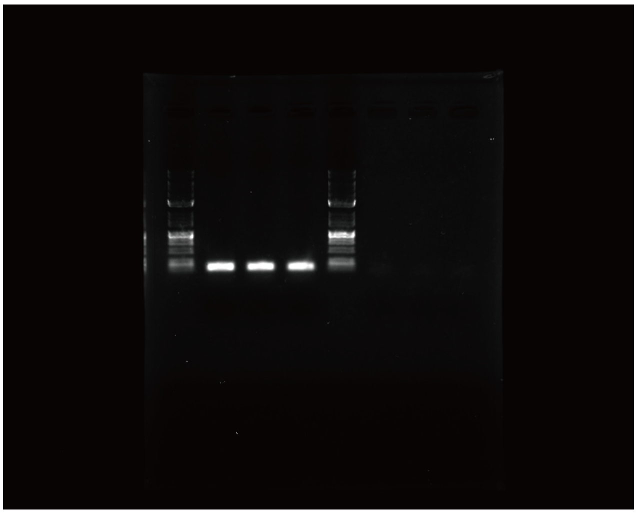

Supplementary Figure 5a

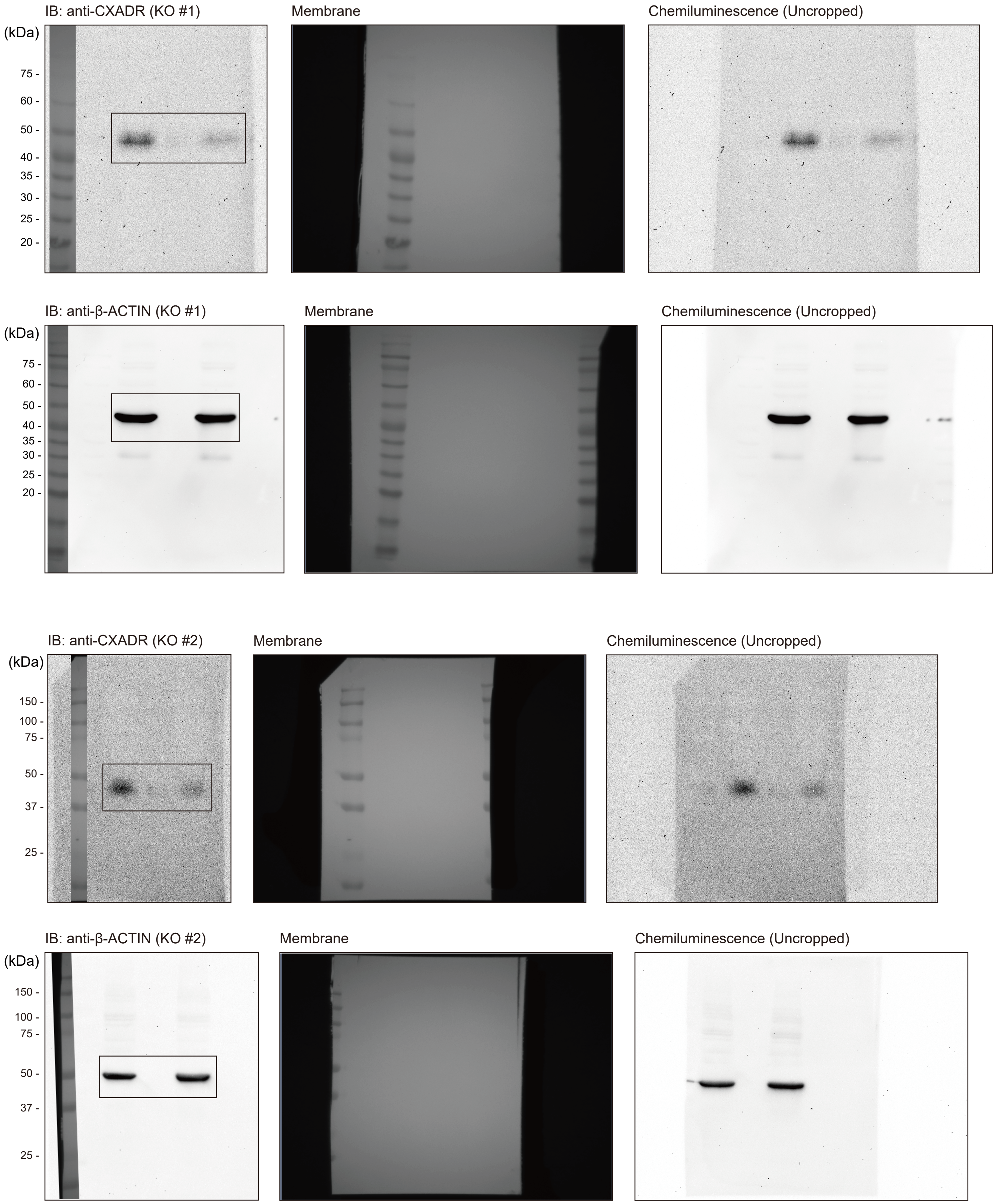

Supplementary Figure 11a

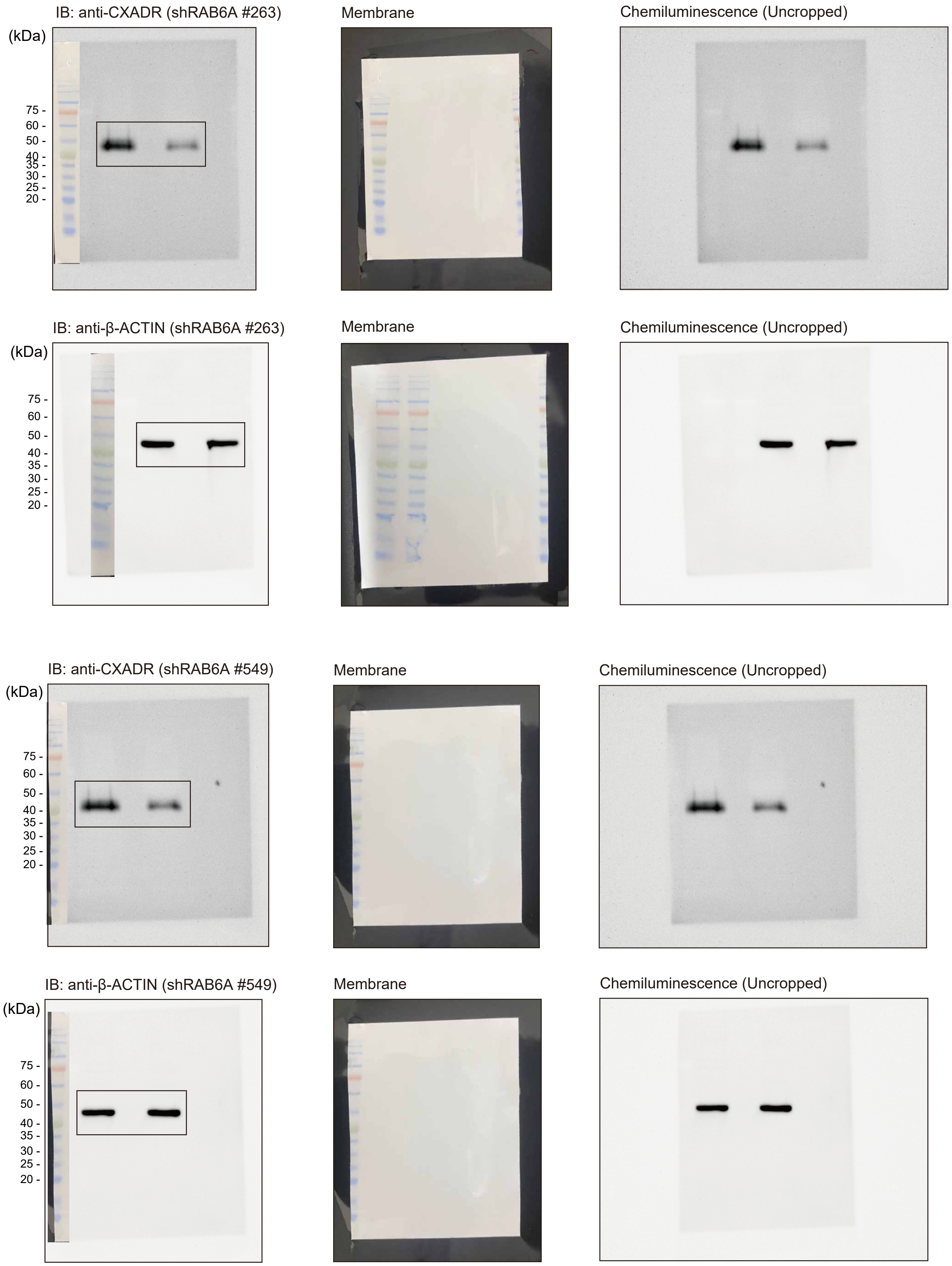

IB: anti-CXADR (shFAM177A1 #186)

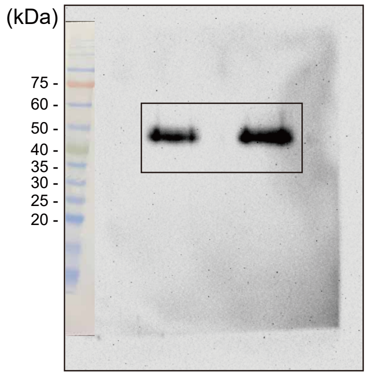

Membrane

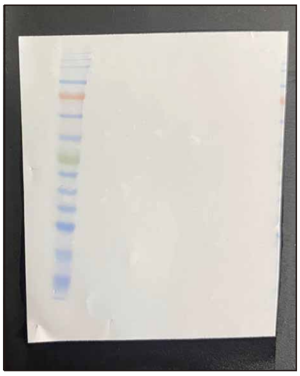

Chemiluminescence (Uncropped)

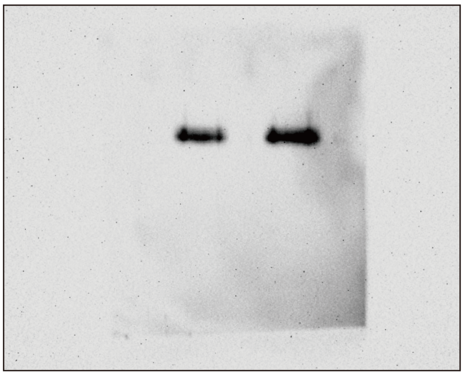

IB: anti-β-ACTIN (shFAM177A1 #186)

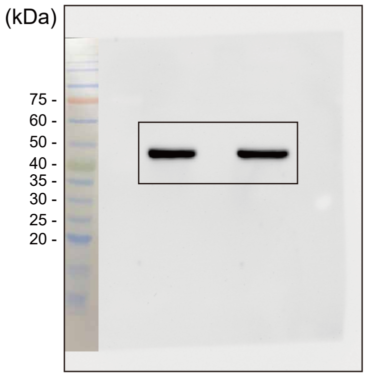

Membrane

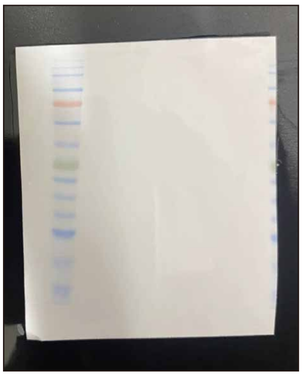

Chemiluminescence (Uncropped)

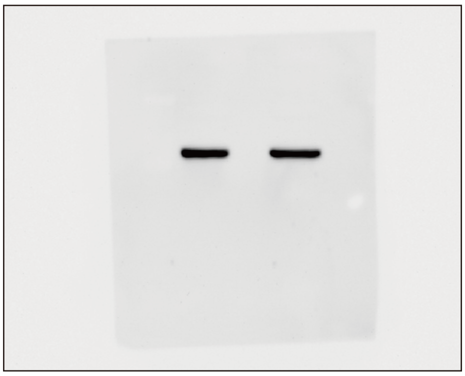

IB: anti-CXADR (shSEC23IP #880)

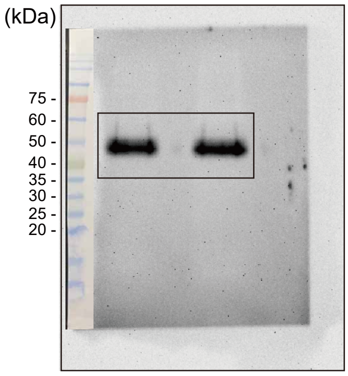

Membrane

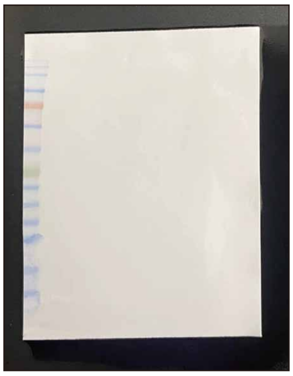

Chemiluminescence (Uncropped)

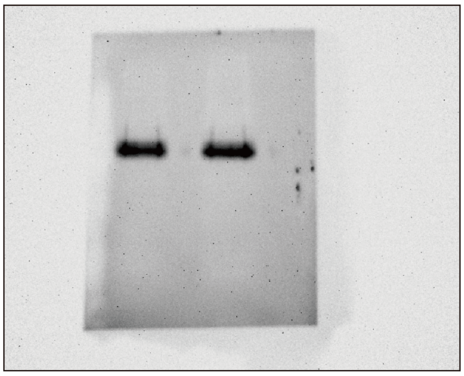

IB: anti-β-ACTIN (shSEC23IP #880)

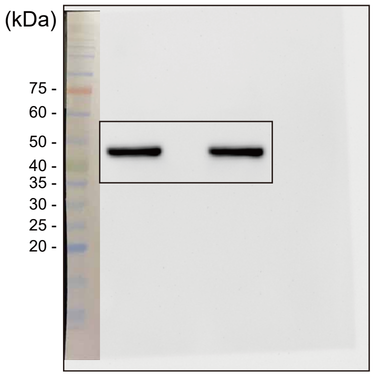

Membrane

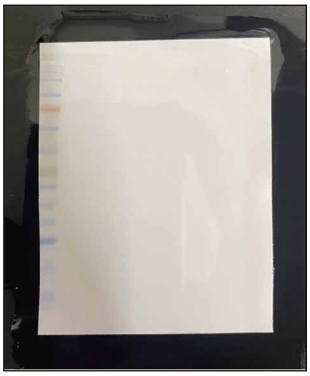

Chemiluminescence (Uncropped)

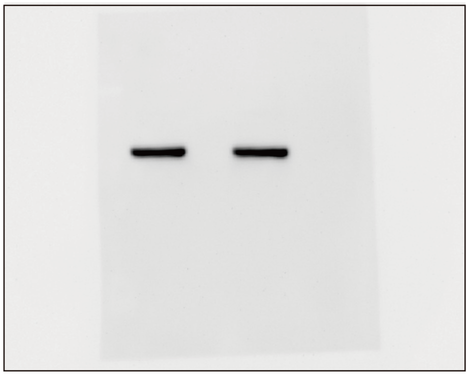

IB: anti-CXADR (shSEC23IP #2941)

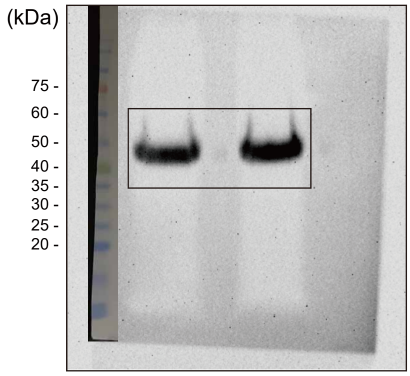

Membrane

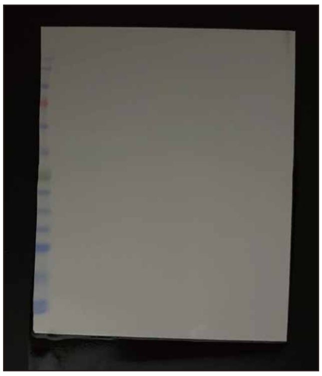

Chemiluminescence (Uncropped)

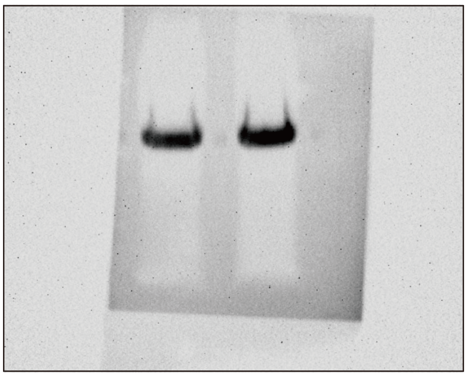

IB: anti-β-ACTIN (shSEC23IP #2941)

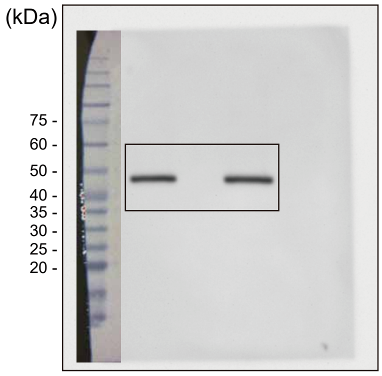

Membrane

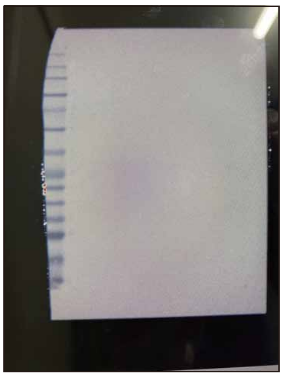

Chemiluminescence (Uncropped)

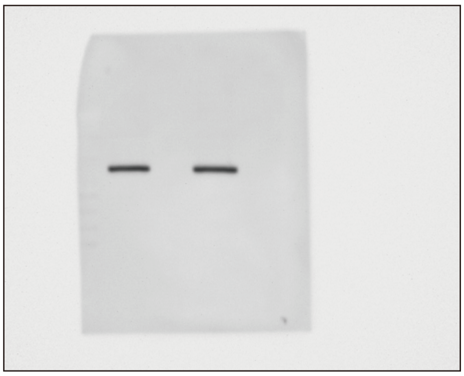

IB: anti-CXADR (shSTX13 #526)

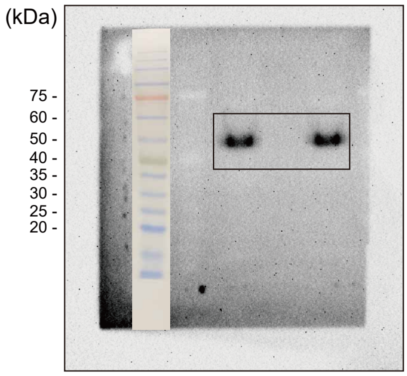

Membrane

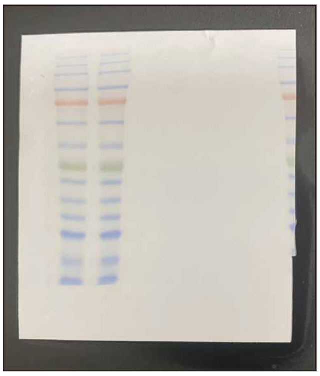

Chemiluminescence (Uncropped)

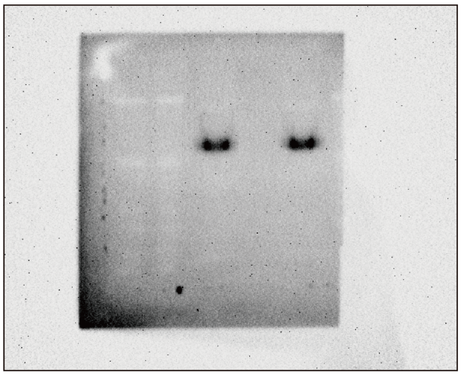

IB: anti-β-ACTIN (shSTX13 #526)

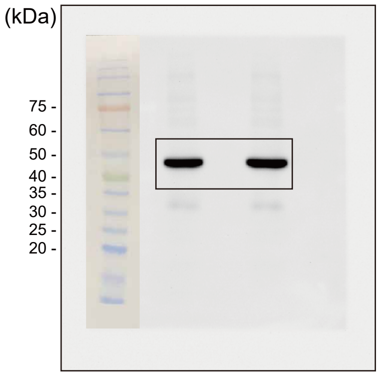

Membrane

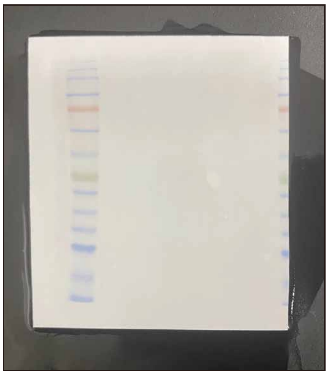

Chemiluminescence (Uncropped)

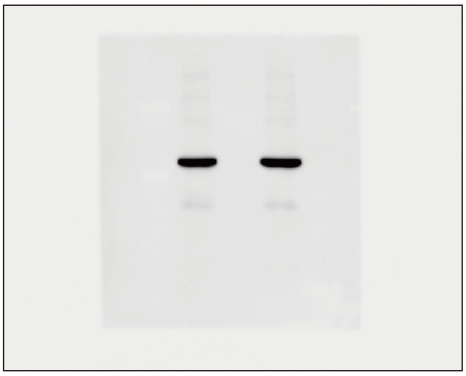

IB: anti-CXADR (shSTX13 #587)

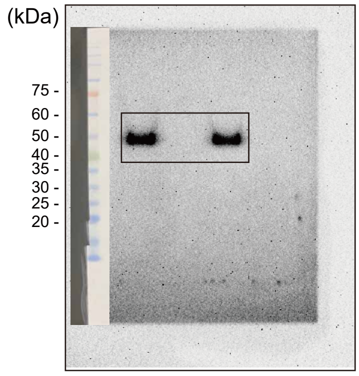

Membrane

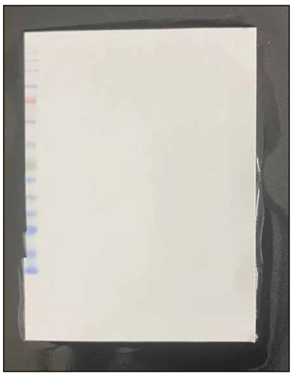

Chemiluminescence (Uncropped)

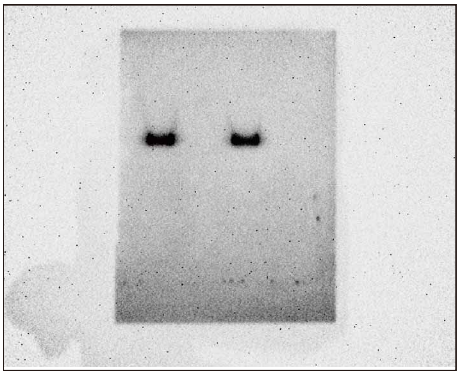

IB: anti-β-ACTIN (shSTX13 #587)

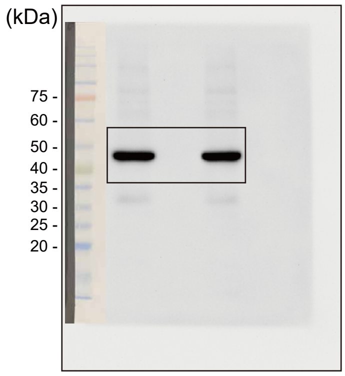

Membrane

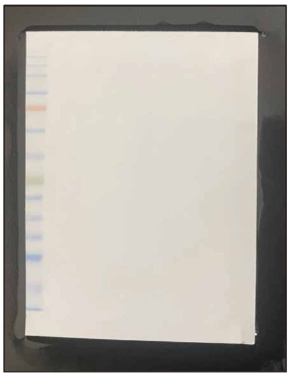

Chemiluminescence (Uncropped)

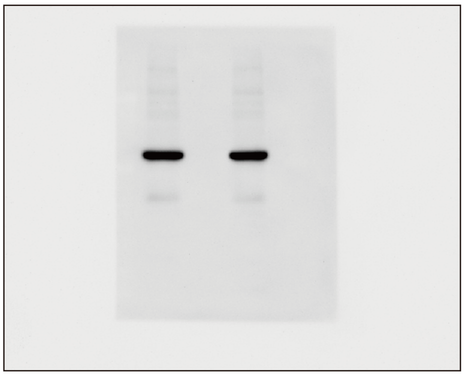

Supplement: Supplementary file 1 — Supplementary Material 1 [file 41598_2026_40840_MOESM1_ESM.pdf]
